# Unrelated umbilical cord blood transplantation for children with hereditary leukodystrophy: A retrospective study

**DOI:** 10.3389/fneur.2022.999919

**Published:** 2022-09-30

**Authors:** Ping Wang, Xiaonan Du, Quanli Shen, Wenjin Jiang, Chen Shen, Hongsheng Wang, Shuizhen Zhou, Yi Wang, Xiaowen Qian, Xiaowen Zhai

**Affiliations:** ^1^Department of Hematology and Oncology, Children's Hospital of Fudan University, Shanghai, China; ^2^Department of Neurology, Children's Hospital of Fudan University, Shanghai, China; ^3^Department of Radiology, Children's Hospital of Fudan University, Shanghai, China

**Keywords:** leukodystrophy, hereditary, umbilical cord blood, transplantation, inherited metabolic disease

## Abstract

**Objective:**

To analyze the efficiency of unrelated umbilical cord blood transplantation (UCBT) in the treatment of hereditary leukodystrophy following busulfan- and cyclophosphamide-based myeloablative chemotherapy.

**Methods:**

A retrospective study was performed in patients with hereditary leukodystrophy who underwent UCBT after myeloablative chemotherapy between April 2015 and March 2020.

**Results:**

The study cohort included 12 pediatric patients (ten males), nine with cerebral adrenoleukodystrophy (ALD) and three with juvenile globoid cell leukodystrophy (GLD). All received HLA-matched or partially mismatched unrelated UCBT. There were no cases of graft rejection. Median neutrophil engraftment time was 20 days [12–33 days] and median platelet engraftment time was 29 days [14–65 days]. Median follow-up was 36 months [1–86 months], and the overall survival rate for patients with cerebral ALD and juvenile GLD after UCBT was 77.8% (7/9) and 100% (3/3), respectively. In patients with ALD, although lipid profiles (serum very-long-chain fatty acid) were improved post-UCBT, six patients demonstrated worse neurologic function score and performance status post-UCBT, and six patients had higher *Loes* scores at last follow-up compared with baseline. In patients with juvenile GLD, all patients showed stable neurologic function score and performance status despite the *Loes* score of one patient increased slightly after transplantation.

**Conclusion:**

In patients with cerebral ALD, patients with no or mild neurological symptoms can benefit from UCBT, while UCBT cannot reverse advanced disease. In patients with juvenile GLD, UCBT is safe and contributes to stabilize neurological function.

## Introduction

Hereditary leukodystrophies are a rare group of inherited metabolic diseases caused by mutations in genes encoding metabolic enzymes or factors leading to abnormal development or diffuse damage to the myelin sheath. Adrenoleukodystrophy (ALD) is an X-linked β-oxidation disorder of very-long-chain fatty acids (VLCFAs) caused by *ABCD1* gene mutations (1). More than 900 pathogenic variants in *ABCD1* associated with ALD have been identified (2). *ABCD1* gene mutations lead to an impaired plasticity of macrophages, and the altered microglia caused pro-inflammatory environment which contributed to the devastating demyelination in cerebral ALD (3). Neurological symptoms include audiovisual deficits, intellectual disability, cognitive impairments, behavioral abnormalities, and neuropsychiatric disorders. There are eight ALD subtypes, of which cerebral ALD in childhood accounts for about 30% of all cases and has the most severe clinical manifestations. After the onset of cerebral ALD, patients may exhibit disability and dementia followed by death in a few months to years (4). Globoid cell leukodystrophy (GLD) is an autosomal recessive genetic disease caused by mutations in *GALC* gene that cause demyelination through lack of galactocerebrosidase activity and ensuing accumulation of β-galactosides and derivatives. More than 270 different mutations in *GALC* related to GLD have been cataloged in the Human Gene Mutation Database (5). Globoid cell leukodystrophy includes four clinical subtypes: early infantile, late infantile, juvenile, and adult phenotype. Patients with the early infantile phenotype are often younger than 6 months old and present with agitation, convulsion, audiovisual deficits, and feeding difficulties; further, the disease progresses rapidly, and median survival is only 2 years (6). Alternatively, patients with the later-onset types present with dyskinesia, visual impairment, mental decline, and seizures (7).

In some developed countries or regions, newborn screening enables early diagnosis and intervention (8, 9). In developing countries like China, however, newborn screening did not include hereditary leukodystrophies, and patients often had neurological deficits before diagnosis, unless there were other probands in their families. Furtherly, early diagnosis was difficult due to the insidious onset, diversity, and non-specificity of symptoms. Dietary control and glucocorticoid administration may alleviate some symptoms, but these measures do not improve neurological deficits. Alternatively, allogeneic hematopoietic stem cell transplantation (allo-HSCT) can slow the progression of neuropathy and promote the long-term survival of children with cerebral ALD and GLD (8, 10–12). In addition, not all kinds of hereditary leukodystrophies are transplantable. Only lysosomal leukodystrophies (GLD and metachromatic leukodystrophy) as well as microgliopathies (ALD and Adult-onset leukoencephalopathy with axonal spheroids and pigmented glia) benefit from HSCT. The benefits of HSCT for GLD are attributed to “cross correction”, a process in which the GALC enzyme secreted by donor cells binds to surface receptors on recipient host cells and is accumulated by pinocytosis, thereby compensating for the enzymatic deficit. If GALC activity is sufficient, demyelination and neuropathy may be prevented (13). In contrast, the therapeutic mechanisms of HSCT for ALD are still unclear. It has been proposed that monocytes from the donor can cross the blood–brain barrier and differentiate into microglia, and that these microglia help restore *ABCD1* activity (14). However, many hereditary leukodystrophy patients lack a matched sibling donor (MSD) of hematopoietic stem cells. Therefore, it is critical that patients without an MSD find a suitable unrelated donor as soon as possible. Unrelated umbilical cord blood stem cells (UCB) which are advantageous due to easy isolation and low HLA compatibility requirements provide an alternative source of hematopoietic stem cells. As hereditary leukodystrophies are relatively rare, there are few case series on the efficacy of unrelated umbilical cord blood stem cell transplantation (UCBT). Here we report the outcomes of twelve consecutive patients receiving unrelated UCBT at a single center to identify the most promising candidates.

## Patients and methods

### Patients

All hereditary leukodystrophy patients treated by unrelated UCBT from April 2015 to March 2020 in the Children's Hospital of Fudan University were included in this study. Diagnosis was based on clinical manifestations, enzyme detection, and neuroimaging examinations, and then confirmed by gene sequencing. All patients were younger than 18 years old and none had an MSD. All guardians provided written informed consent before transplantation and the study was approved by the ethics committee of the Children's Hospital of Fudan University (2016-162).

### UCBT data and conditioning regimen

Unrelated UCB was provided by the Chinese Cord Blood Bank with HLA matching at a minimum of 7/10 loci according to high-resolution typing identity of A, B, Cw, DRB1, and DQ. The median total nucleated cell (TNC) infused dose was 4.9 × 10^7^/kg (range, 3.0 × 10^7^-15.3 × 10^7^/kg), and the median CD34^+^ cell dose was 1.55 × 10^5^/kg (range, 0.37 × 10^5^-19.95 × 10^5^/kg). The conditioning regimen included intravenous busulfan (Bu) at a dose of 0.8–1 mg/kg given intravenously (i.v.) every 6 h for 4 days (total dose, 12.8–16 mg/kg), fludarabine (Flu) at 30 mg/m^2^/d by i.v. for 5 days (total dose, 150 mg/m^2^), and cyclophosphamide (CY) at 50 mg/kg/d by i.v. for 2 days (total dose, 100 mg/kg). Rabbit anti-human thymocyte immunoglobulin (ATG [Genzyme], 5 mg/kg) was used in ten patients. Oral tacrolimus (FK506) was administered starting 4 days before transplantation to prevent graft vs. host disease (GvHD). The target concentration of FK506 was 5–10 ng/mL. In addition to FK506, mycophenolate mofetil (MMF) was administered to the patients who received UCB with 7/10 HLA matching on the first day post-transplantation.

### Chimeric monitoring and engraftment definition

Donor-recipient chimerism was tested using peripheral blood leukocytes by the short tandem repeat technique (15) at 2 weeks, 1 month, 2 months, 3 months, 6 months, 9 months, and 1 year after transplantation. More than 95% donor-derived cells were defined as complete donor chimerism. An absolute neutrophil count >0.5 × 10^9^/L for 3 consecutive days was defined as neutrophil engraftment, and platelet count >20 × 10^9^/L for 7 consecutive days without platelet transfusion was defined as platelet engraftment.

### Neurological function scores, performance status, and *Loes* scores

Each patient was assigned a Neurologic Function Score (NFS) pre- and post-UCBT based on evaluations of vision, hearing, communication, swallowing, urinary and fecal control, movement, and the presence of afebrile convulsions (16). Performance status (PS) was scored by the Lansky standard (17). In addition, a *Loes* score of cranial magnetic resonance images (MRI) lesion severity was assigned by a senior radiologist (18, 19).

### Supportive treatment

All patients were cared for in an independent laminar flow ward before neutrophil engraftment. Ganciclovir (10 mg/kg/day, from the beginning of conditioning to day−1 pre-UCBT) and acyclovir (750 mg/m^2^/day, from day 0 to day +270 post-UCBT) were used to prevent virus infection, caspofungin (50 mg/m^2^/day, from the beginning of conditioning to neutrophil engraftment) and voriconazole (16 mg/kg/day from neutrophil engraftment to day +180) were used to prevent fungal infection, and sulfamethoxazole (25 mg/kg/day 2 days per week, from neutrophil engraftment to 6 months after immunosuppressant discontinuation) was used to prevent *Pneumocystis carinii* infection. Patients received intravenous immunoglobulin (500 mg/kg/dose) every 2 weeks starting on day +1 post-UCBT and continuing until B-lymphocyte count surpassed 200/μL.

### Statistical analysis

Descriptive statistics were presented for baseline characteristics, outcomes post-transplant and the most recent follow-up. Survival curves were estimated by the Kaplan-Meier method, and log-rank test was used for comparison. All statistical analyses were performed with GraphPad Prism 8.

## Results

### Clinical conditions before transplantation

Twelve patients with hereditary leukodystrophy were treated by UCBT. Clinical and demographic characteristics of these patients (p1–p12) are summarized in Table 1. Nine (p1–p9 in Table 1) were diagnosed with cerebral ALD and harbored unique maternally inherited *ABCD1* mutations (mainly point mutations in exons 1 and 3). Two patients (p10 and p12) were diagnosed with juvenile type GLD and harbored inherited *GALC* gene mutations. The remaining one (p11) was the sibling of p10, carried the same *GALC* gene mutation and had a reduced leukocyte galactocerebrosidase activity, while her brain MRI showed normal before transplantation. The median onset age was 7 years (range, about 3–8 years). The median age of these patients at the time of diagnosis was 7 years (range, about 0.4–12.7 years). Ten patients (except p3 with ALD and p11 with GLD) had neurological symptoms before transplantation, eleven patients (except p11) had abnormal white matter signals on cranial MRI before transplantation, and seven patients (p1, p3, and p5–p9) had adrenal cortex dysfunction and were receiving glucocorticoid replacement therapy. Serum VLCFAs were elevated in all ALD patients and leukocyte galactocerebrosidase activity was reduced in all GLD patients.

**Table 1:** Baseline clinical characteristics of the 12 patients with heredity leukodystrophy.

**Patient**	**Sex**	**Onset/** **Diagnosis age (years)**	**Gene affected**	**Mutations**	**Nervous system symptom**	**Lesions on cranial MRI**	**Adrenocortical insufficiency**	**VLCFAs**	**Galacto** **-cerebrosidase (nmol/17h/mg)**
1	Male	8.3/8.6	ABCD1	exon1 c.829G>A, p.G277R	+	+	+	↑	NA
2	Male	7.0/7.6	ABCD1	exon1 c.593G>A, p.T198K	+	+	–	↑	NA
3	Male	about 3/5.6	ABCD1	exon3 c.1552C>T, p.R518W	–	+	+	↑	NA
4	Male	5.2/5.5	ABCD1	c.1992-2A>G, p?	+	+	–	↑	NA
5	Male	about 8/12.7	ABCD1	exon1 c.465delG, p.G156Afs*42	+	+	+	↑	NA
6	Male	4.0/4.9	ABCD1	exon1 c.650A>C, p.K217T	+	+	+	↑	NA
7	Male	6.3/6.4	ABCD1	exon 3–10 del	+	+	+	↑	NA
8	Male	7.2 8.2	ABCD1	exon 1 c.529C >T, p.Q177X	+	+	+	↑	NA
9	Male	7.7/8.0	ABCD1	exon 10 c.2006A>G, p.H669R	+	+	+	↑	NA
10	Female	7.5/7.7	GALC	exon 8 c.812G>A, p.W271X het	+	+	NA	NA	4.76↓ (12.89–100.93)
				exon 1 c.136G>T, p.D46Y het					
11	Female	–/0.4	GALC	exon 8 c.812G>A, p.W271X het	–	–	NA	NA	6.47↓ (12.89–100.93)
				exon 1 c.136G>T, p.D46Y het					
12	Male	4.3/4.5	GALC	exon 10 c.1090T>G, p.L364V het	+	+	NA	NA	2.11↓ (12.89–100.93)
				exon 1 c.136G>T, p.D46Y het					

NA, not applicable; MRI, magnetic resonance imaging; VLCFA, very-long-chain fatty acids; ↑, higher than reference range; ↓, lower than reference range.

### Transplantation, chimerism and hematopoietic reconstitution Post-UCBT

The median age at UCBT was 7.2 years (range, 0.8–12.9 years) and median body weight at UCBT was 24.3 kg (range, 8.5–38 kg). See Table 2 for details of each basic characteristics of transplantation procedure. All patients demonstrated complete donor chimerism (CDC) by day +14, and the chimerism was stable during follow-up. The median neutrophil engraftment time was 20 days (range, 12–33 days) and the median platelet engraftment time was 29 days (range, 14–65 days) after transplantation.

**Table 2:** Basic characteristics of each umbilical cord blood stem cell transplantation procedure.

**Patient**	**UCBT age (years)**	**UCBT weight (kg)**	**HLA matching**	**TNC/kg × 10^7^**	**CD34^+^/kg × 10^5^**	**Conditioning regimen**	**GvHD prophylaxis**
1	8.8	38	8/10	5.2	1.25	BU/FLU/CY	FK506
2	7.8	25	10/10	3.4	0.37	BU/FLU/CY	FK506
3	6.2	24	8/10	4.4	0.57	ATG+BU/FLU/CY	FK506
4	5.5	17.5	8/10	7.2	1.34	ATG+BU/FLU/CY	FK506
5	12.9	36	7/10	3.8	1.22	ATG+BU/FLU/CY	FK506/MMF
6	5.0	20	8/10	6.4	3.20	ATG+BU/FLU/CY	FK506
7	6.5	23.5	8/10	5.2	0.82	ATG+BU/FLU/CY	FK506
8	8.2	24.5	8/10	4.6	3.29	ATG+BU/FLU/CY	FK506
9	8.2	38	9/10	3.0	1.76	ATG+BU/FLU/CY	FK506
10	8.0	22	10/10	5.3	7.32	ATG+BU/FLU/CY	FK506
11	0.8	8.5	9/10	15.3	19.95	ATG+BU/FLU/CY	FK506
12	4.5	24.5	8/10	3.7	3.22	ATG+BU/FLU/CY	FK506

UCBT, umbilical cord blood stem cell transplantation; HLA, human leukocyte antigen; ATG, antithymocyte globulin; Bu, Busulphan; Cy, Cyclophosphamide; Flu, Fludarabine; MMF, mycophenolate mofetil.

### Prognosis and transplant-related complications

The median follow-up time after UCBT was 36 months (range, 1–86 months). Of the 12 patients treated, two died (both with ALD), one of severe pneumonia complicated by sepsis within one month after UCBT and the other of severe pneumonia due to rapid progression of neurological symptoms 5 months after UCBT, for an overall survival rate of 81.8%. Four patients (33.3%) developed grade II acute GvHD, and all responded to methylprednisolone therapy. There were no cases of chronic GvHD. Half of the patients developed pulmonary infection after transplantation, including the two fatalities. Four cases developed cytomegalovirus (CMV) viremia, all of which responded to ganciclovir and/or foscarnet as confirmed by negative CMV-DNA tests. Two patients (16.7%) developed delayed hemorrhagic cystitis caused by BK virus infection, and both cases gradually eased after hydration, dieresis, and indwelling catheterization. Two patients (16.7%) developed urinary tract infections after transplantation that were successfully treated with sensitive antibiotics. Finally, one male patient developed autoimmune hemolytic anemia after transplantation that was controlled by plasma exchange, rituximab, and methylprednisolone. However, neurological symptoms deteriorated rapidly. See Table 3 for details of post-UCBT complications and treatments. The Kaplan-Meier overall survival curves for patients with ALD or GLD are shown in Supplementary Figure 1.

**Table 3:** Outcomes and complications of UCBT.

**Patient**	**Engraftment**	**Chimerism**	**Neutrophil engraftment day**	**Platelet engraftment day**	**GvHD**	**Complication**	**Outcome (months post-UCBT)**
1	Engrafted	CDC	19	22	I	Pulmonary fungal infection	Alive (86)
2	Engrafted	CDC	32	–	–	Severe pneumonia, septic shock	Died (1)
						Gastrointestinal hemorrhage	
3	Engrafted	CDC	33	37	I	None	Alive/well^#^ (54)
4	Engrafted	CDC	21	26	II	Urinary tract infection	Alive (48)
						Pneumonia, CMV viremia	
5	Engrafted	CDC	22	43	II	Severe pneumonia (*Klebsiella pneumoniae, Candida tropical*)	Died (5)
						Septic shock	
						CMV viremia	
6	Engrafted	CDC	12	14	I	Severe pneumonia (*Acinetobacter baumannii*)	Alive (30)
						CMV viremia	
						AIHA	
7	Engrafted	CDC	18	29	II	Hemorrhagic cystitis	Alive (39)
8	Engrafted	CDC	24	35	I	None	Alive (33)
9	Engrafted	CDC	19	29	II	Hemorrhagic cystitis,	Alive (31)
						Pneumonia	
10	Engrafted	CDC	16	17	I	Urinary tract infection	Alive (54)
11	Engrafted	CDC	25	37	I	None	Alive/well^#^ (45)
12	Engrafted	CDC	14	65	I	CMV viremia	Alive (28)

AIHA, autoimmune hemolytic anemia; CDC, complete donor chimerism;

# Nervous system asymptomatic.

### Comparison of neurologic function score, performance status, and *Loes* Score Pre-UCBT and Post-UCBT

Neurologic function, PS, and *Loes* scores were evaluated regularly before and after transplantation. The censored follow-up time for the patients was over 24 months. Neurologic outcome can only be evaluated in eleven patients (all except p2). The Kaplan-Meier overall survival curves for NFS-“stable” or NFS-“unstable” patients are shown in Supplementary Figure 2.

**Figure 1:**
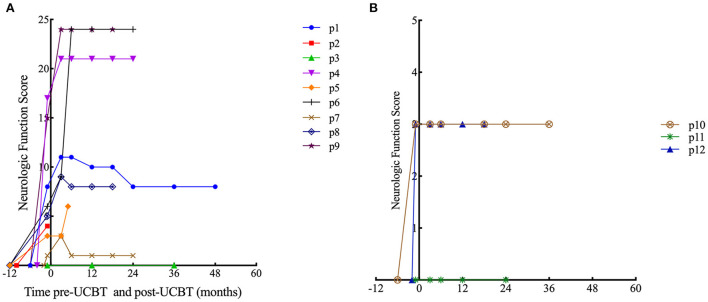
Time course of Neurologic Function Score changes. **(A)** Patients with ALD. **(B)** Patients with GLD.

**Figure 2:**
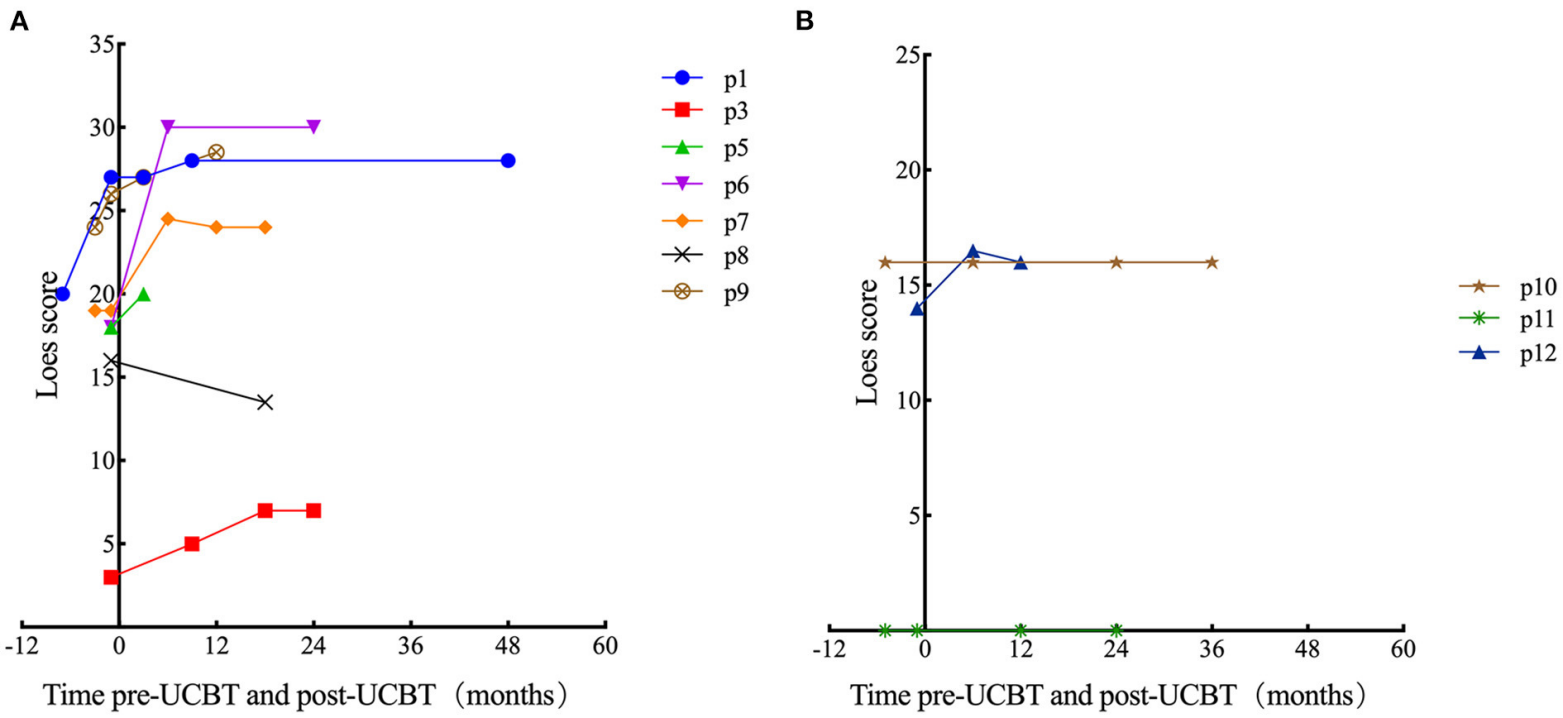
Time course of *Loes* score changes for all patients. In some cases, there was no post-UCBT data because of early death (p2) or because the parent (of p4) refused MRI examination after transplantation. **(A)** Patients with ALD. **(B)** Patients with GLD.

In patients with ALD, eight patients (all except p3) demonstrated neurological impairments of varying severity before transplantation (NFS range, 1–17 points), all the eight patients showed varying degrees of neurologic symptom aggravation within 6 months after UCBT (NFS range, 3–24 points) and then gradually stabilized. Six patients (all except p1, p3, and p7) demonstrated worse NFS at last follow-up compared with the baseline. In patient 6, neurologic symptoms deteriorated markedly after transplantation (6 points before transplantation to 9 points 3 months after transplantation to 24 points 6 months after transplantation) concomitant with severe autoimmune hemolytic anemia, and there was no significant recovery during follow-up. Among the surviving seven patients (p1, p3, p4, p6–p9), the NFS of patient 1 and patient 7 was similar to pre-UCBT baseline at last follow-up (Figure 1A for details). Three patients (p3, p7, and p8) showed the same performance status as before transplantation at the last follow-up after UCBT. The remaining six patients demonstrated different degrees of PS deterioration including two patients who deceased after UCBT (Table 4). All patients with ALD demonstrated different degrees of brain damage before transplantation as measured by *Loes* scoring of MRI, and most (except p8) exhibited further increases in brain lesion severity after UCBT with stabilization by 9 months post-treatment (Figure 2A). Noteworthily, patient 7 had a relatively high *Loes* score (19 points) before transplantation, while the NFS was relatively low (1 point), and he obtained the great benefit after transplantation (NFS 1 point) even though the *Loes* score increased (24 points) post-UCBT. The pre- and post-UCBT MRI of patients with ALD are shown in Supplementary Figures 3–11.

**Table 4:** Performance status scores of patients with heredity leukodystrophy pre-UCBT and post-UCBT.

**Patient**	**PS pre-UCBT**	**PS post-UCBT^&^**
1	70	50
2	80	0
3	100	100
4	50	40
5	60	0
6	50	40
7	100	100
8	80	80
9	50	40
10	80	80
11	100	100
12	80	80

Performance status scores (PS) by Lansky (17);

& at last follow-up.

In patients with GLD, two patients (p10 and p12) demonstrated neurological impairments before transplantation (NFS both 3 points). Two female patients (p10 and p11) with GLD were siblings. The neurological symptoms of the elder sibling (p10) barely progressed after transplantation as evidenced by stable NFS and PS (Figure 1B and Table 4 for details). Fortunately, the younger sibling (p11) had no imaging lesions or nervous system involvement before or after UCBT. Another male (p12) with GLD was also neurologically stable before and after UCBT as evidenced by stable NFS and PS, although the *Loes* score increased slightly after transplantation. The changes in *Loes* scores for patients with GLD throughout the treatment period are shown in Figure 2B. The pre- and post-UCBT MRI of patients with GLD are shown in Supplementary Figures 3–11.

### Changes in lipid metabolism

**Figure 3:**
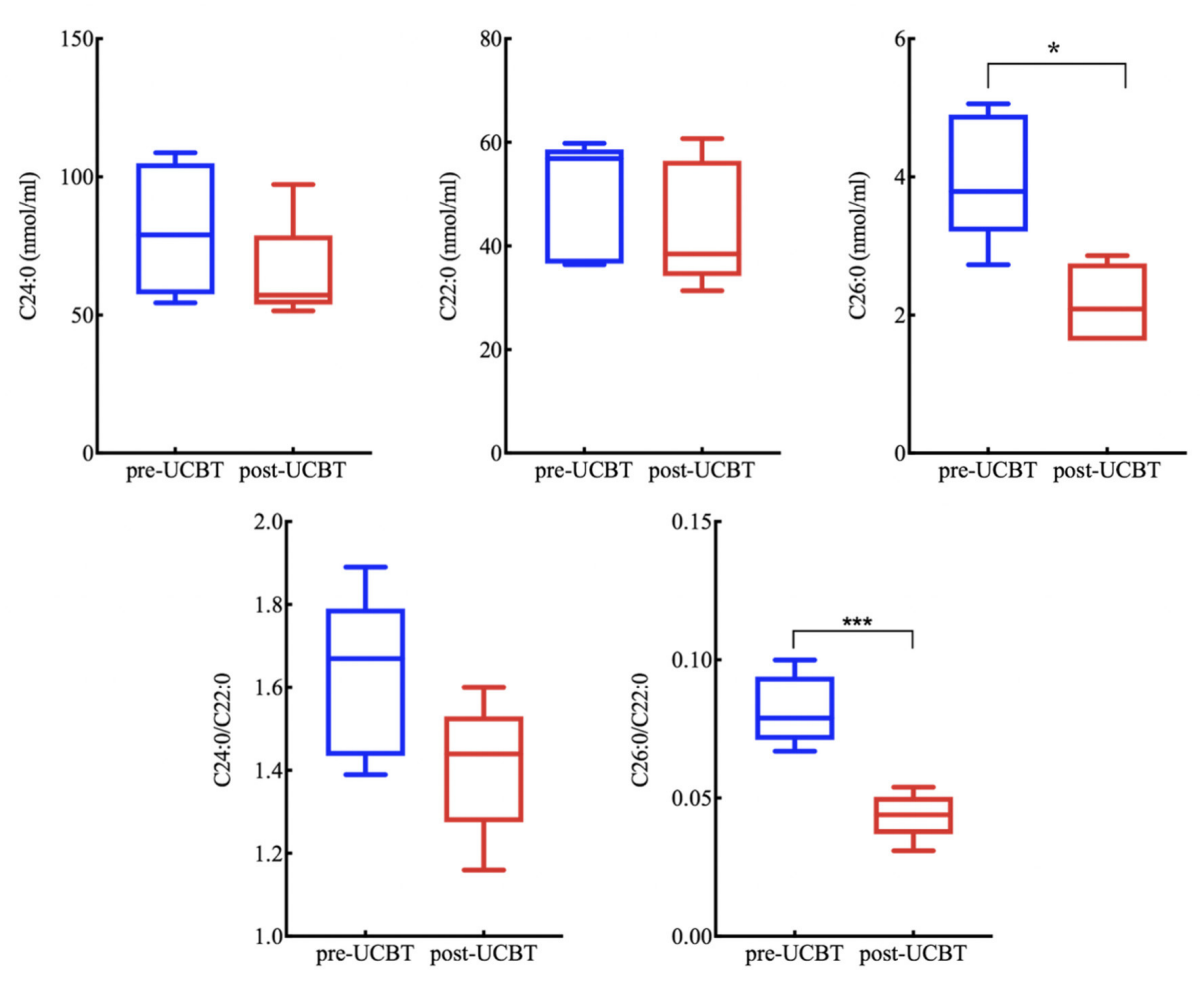
Serum very-long-chain fatty acid (VLCFA) concentrations for all ALD patients (excluding p2, p4, and p5 without post-UCBT data) were reduced 1 year post-UCBT. (Reference value C22:0 ≤ 96.3 nmol/mL, C24:0 ≤ 91.4 nmol/mL, C26:0 ≤ 1.30 nmol/mL, C24:0/C22:0 ≤ 1.39, C26:0/C22:0 ≤ 0.023). * means *P* < 0.05. *** means *P* < 0.001.

The serum VLCFA concentrations of most surviving ALD patients (except p4) were measured regularly following treatment. Both absolute C26:0 concentration and the C26:0/C22:0 ratio were significantly reduced 1 year post-UCBT (Figure 3), indicating partial restoration of *ABCD1* activity.

## Discussion

Treatment of hereditary leukodystrophy is limited by a lack of effective drugs. Diet adjustment and glucocorticoids may alleviate some symptoms, but cannot improve neuropathy, which is the predominant cause of functional impairment and frequently an indirect cause of death from infection. Currently, hematopoietic stem cell transplantation is the only way to prevent the progression of neuropathy and prolong survival. Cerebral type ALD and GLD in childhood are both indications for HSCT (11). While ALD patients with genetic diagnosis but absent symptoms should instead receive regular MRI and neurological examinations to assess myelination status. When the patient is clinically presymptomatic, but cerebral disease just begins as revealed by the cranial MRI, the patient needs to transplant as early as possible to prevent further progression and improve prognosis (20).

The neuropathy associated with childhood cerebral ALD and GLD progresses rapidly, so timely transplantation is critical. The European Society for Blood and Marrow Transplantation recommends related donors as the first choice for stem cell transplantation, followed by unrelated donors with at least 4/6 site matching. UCB is easy to obtain and rich in stem cells (13). However, the failure rate of UCB engraftment is higher than that of bone marrow or peripheral blood stem cells. As recently reported, for cerebral ALD patients who received unrelated UCBT with reduced intensity conditioning or myeloablative conditioning, the engraftment rates were 64.3% (9/14) and 73.9% (17/23), respectively (21, 22). Another study reported on the use of a myeloablative conditioning with UCB from unrelated donors for treatment of infantile GLD, with favorable engraftment (23). In the present study, twelve patients were treated with BU/CY- based myeloablative conditioning and all demonstrated complete donor cell chimerism 2 weeks after transplantation that remained stable during follow-up. Our study emphasized the priority of myeloablative conditioning in patients with cerebral ALD or juvenile GLD who underwent UCBT.

At present, hereditary leukodystrophies are not part of newborn screening in China. In most patients, the initial symptoms are non-specific, so diagnosis is often delayed, resulting in progression of neuropathy before transplantation. In our patients, eight patients with cerebral ALD and two patients with juvenile GLD had nervous system symptoms before transplantation. The median time from onset to UCBT was 6 months (2–58 months). Both *Loes* and NFS scores indicated substantial structural and functional neurological impairment before transplantation. Although neurological function was generally more stable following transplantation, damage that had occurred before transplantation was largely irreversible, underscoring the urgency of diagnostic confirmation and treatment.

In previous studies, the overall survival rates of cerebral ALD following UCBT ranged from 59% to 90%, and the analysis of prognostic factors showed that the early-stage of disease, fewer and less extensive brain imaging lesions, and sufficient stem cells were all predictive of better prognosis (11, 16, 21, 22, 24). In the present study, the overall survival rate of patients with cerebral ALD was 77.8%. The latest guideline recommended that patients with early-stage cerebral ALD (NFS <2 and *Loes* score <10) should undergo stem cell transplantation. On the contrary, individuals with advanced cerebral ALD were recommended for natural history studies or clinical trials in consideration of high risk of transplant-related mortality and few neurologic benefits (25). In our study, only two patients were in the early stage of cerebral ALD, and both achieved stable neurological function after UCBT. In addition, the remain seven patients with advanced cerebral ALD also received UCBT under the background of one-child policy which was operated between 1980 and 2016 in China. Despite the significant risks of HSCT, no other effective therapeutic option existed for them. The parents/guardians were fully informed of the possible adverse prognosis of transplantation, and they still insist on undergoing transplantation to save the life of their only child. It should be noted that HSCT belonged to compassion use in this circumstance.

Generally, the prognosis is better for juvenile GLD compared to infantile subtype GLD. However, juvenile onset patients often have less classic presentations, making diagnosis difficult and delayed (26). Infantile GLD diagnosed with a family history or by newborn screening should rapidly proceed to transplant, while infants diagnosed because of clinical symptoms are not recommended for HSCT for the reason of rapid progression (25). Asymptomatic infantile GLD newborns can obtain 100% engraftment and 100% survival through UCBT, and the myelin gradually forms and development skills gradually improve after transplantation (27). Yoon et al. found that HSCT not only prolonged the lifespan but also improved the functional abilities including cognitive and language function, gross and fine motor development for patients with infantile GLD (28). Evidence for improved survival in postsymptomatic transplanted patients with GLD had also been reported by Langan et al. (29). Transplantation of juvenile type GLD patients is less than that of infantile subtype. However, some reports have indicated that patients with juvenile GLD can derive benefit from the procedure (30, 31). In the present study, all patients with juvenile GLD survived and had stable neurological function after UCBT. Our results support the active transplantation in juvenile type GLD to stabilize the neurological function.

GvHD and infection were common complications following transplantation. Compared to stem cells from bone marrow or peripheral blood, the immunogenicity of UCB is lower, so the incidence of severe GvHD after UCBT is relatively reduced (only about 20% to 35% in previous reports) (11). In the current study, there were no cases of chronic or severe GvHD, only four cases of acute grade II GvHD responsive to glucocorticoid therapy. Children with hereditary leukodystrophy may have weak cough reflexes and urinary incontinence due to neuropathy as well as poor immune function after pretreatment, which in combination can increase the risks of pulmonary and urinary tract infections. Indeed, previous studies have found that severe infection associated with disease progression after transplantation is the main cause of death among children with hereditary leukodystrophy (11). In the present study, six of twelve patients developed pulmonary infection after transplantation, and two died, while another four developed CMV viremia. Fortunately, these CMV viremia cases were cured by ganciclovir or foscarnet treatment. In addition, the two cases of urinary tract infection and the two cases of hemorrhagic cystitis were improved by antibiotic treatment or support therapy. Therefore, clinical outcome following UCBT for hereditary leukodystrophy may be improved by more intensive nursing care to prevent pulmonary infection.

Multiple factors can affect the prognosis of neurological function in hereditary leukodystrophy patients after transplantation, including disease severity before transplantation, donor chimerism level after transplantation, GvHD severity, and other transplantation related complications, especially pulmonary infection. At present, most studies on prognosis have focused on survival rate, while a few studies have conducted longitudinal assessment of nerve function. Chiesa et al. found that the stabilization of neurologic function after HSCT was greater in the early disease group vs. advanced disease group in patients with ALD (22). Peters et al. found that only 16% of cerebral ALD patients with neurological deficits before transplantation demonstrated improvement after transplantation, while 56% of patients without neurological deficits before transplantation had no neurological deficits following transplantation (32). Allewelt et al. found that patients with early infantile GLD who underwent HSCT at <30 days of age, had a more favorable outcomes, particularly in domains of mobility, communication, and feeding (23). Van den Broek et al. found that 50% of hereditary leukodystrophy patients with a mild decline in functional status score before transplantation remained stable after transplantation, while 50% exhibited neurological deterioration after transplantation (11). In such cases, disease progression may occur due to enzyme insufficiency before substantial engraftment.

The *Loes* score for cranial MRI can assist in the objective assessment of the lesion severity. Kato et al. found that the *Loes* score would stabilize or improve after HSCT for patients with ALD who were without internal capsule involvement (21). In our study, only patient 3 with ALD showed normal image of internal capsule. He had normal neurological function and the *Loes* score stabilized 18 months after UCBT. In addition, patient 7 with cerebral ALD had a relatively high *Loes* score (involvement of the parietooccipital and frontal white matter, corpus callosum, visual pathway, auditory pathway, internal capsule, basal ganglia, and brain stem) before transplantation, while neurological function score was relatively low (NFS = 1) with no audiovisual dysfunction, and this patient obtained the greatest benefit after transplantation (NFS = 1). Thus, we speculate that neurological function may be the predominant predictive factor for outcome of HSCT rather than *Loes* score.

## Conclusions

Unrelated UCBT is more effective for cerebral ALD patients with mild or no neurological symptoms. Therefore, early diagnosis and timely treatment prior to substantial progression are critical. In contrast, the potential value of UCBT for cerebral ALD patients with rapid disease progression and severe neurological impairment is limited, so parental expectations should be carefully managed. In the condition of no effective therapy for patients with advanced cerebral ALD, only if the purpose is to extend the patients' lifespans, rather than improve neurological function, they can participate in the transplant procedure. Moreover, parents/guardians should be fully informed of the risk of morbidity and mortality associated with the treatment. UCBT is safe and contributes to stabilize neurological function in juvenile GLD patients.

## Data availability statement

The datasets generated and/or analyzed during the current study are available from the corresponding author on reasonable request.

## Ethics statement

The studies involving human participants were reviewed and approved by Ethics Committee of the Children's Hospital of Fudan University. Written informed consent to participate in this study was provided by the participants' legal guardian/next of kin.

## Author contributions

XZ and XQ designed this study. HW, SZ, YW, and XZ provided administrative support. WJ, SZ, YW, and XQ participated in patients' management. PW, XD, QS, and CS collected and assembled data. PW, XD, QS, WJ, CS, and HW analyzed and interpreted data. PW and XD wrote the article. All authors contributed to the article and approved the submitted version.

## Funding

The study was supported by Shanghai Municipal Committee of Science and Technology (21Y31900302) and National Multidisciplinary Cooperative Diagnosis and Treatment Capability Construction Project for Major Diseases.

## Conflict of interest

The authors declare that the research was conducted in the absence of any commercial or financial relationships that could be construed as a potential conflict of interest.

## Publisher's note

All claims expressed in this article are solely those of the authors and do not necessarily represent those of their affiliated organizations, or those of the publisher, the editors and the reviewers. Any product that may be evaluated in this article, or claim that may be made by its manufacturer, is not guaranteed or endorsed by the publisher.
