# Supplementary material for: Unrelated umbilical cord blood transplantation for children with hereditary leukodystrophy: A retrospective study
Source: Front Neurol. 2022 Sep 30;13:999919. doi: 10.3389/fneur.2022.999919 (PMC9561100; doi:10.3389/fneur.2022.999919)
Supplement: Supplementary file 1 [file Data_Sheet_1.docx]

Supplementary Material

# Supplementary Figures


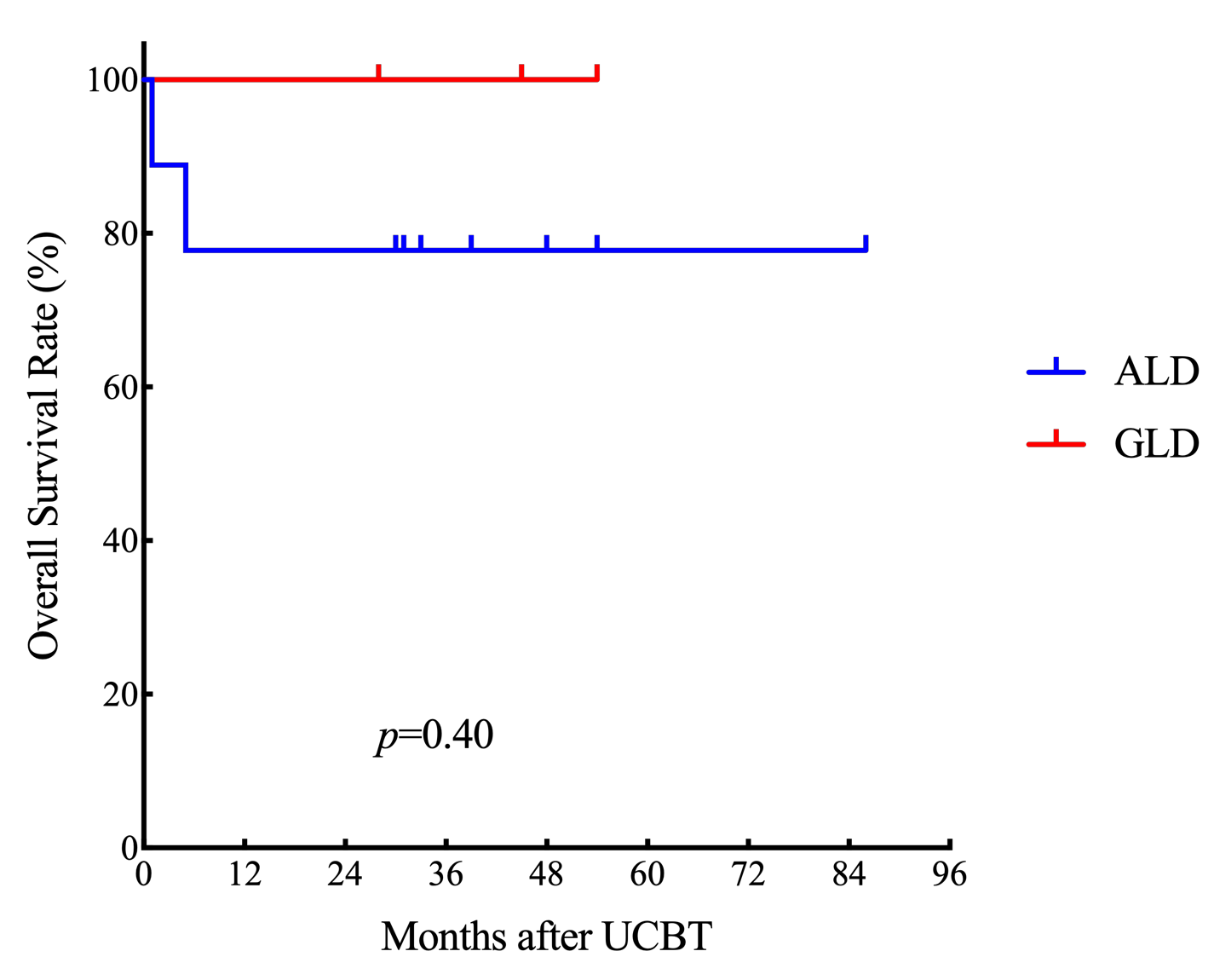


**Supplementary Figure 1.** Kaplan-Meier overall survival curves for patients with ALD or GLD


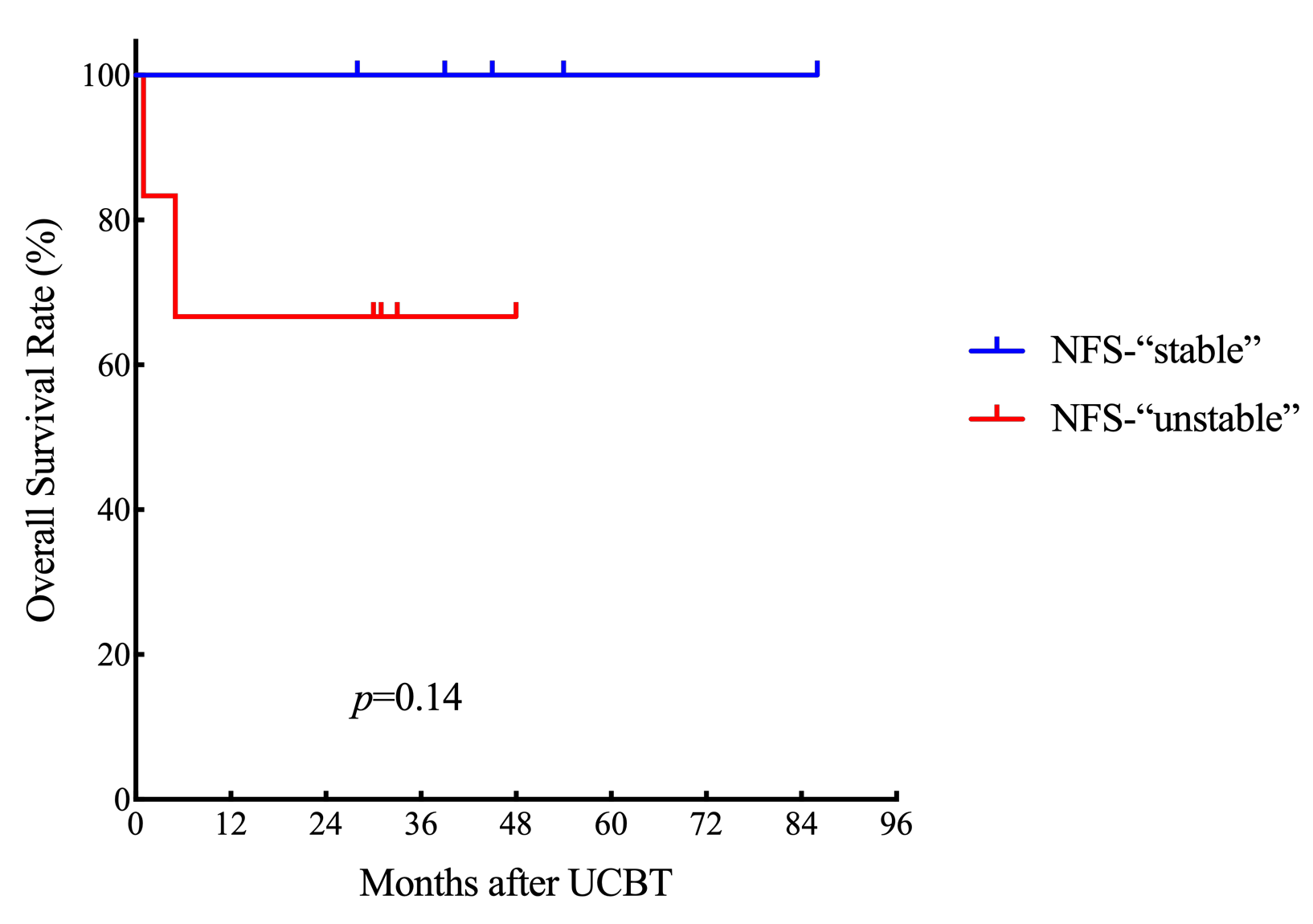


**Supplementary Figure 2.** Kaplan-Meier overall survival curves for NFS-“stable” or NFS-“unstable” patients


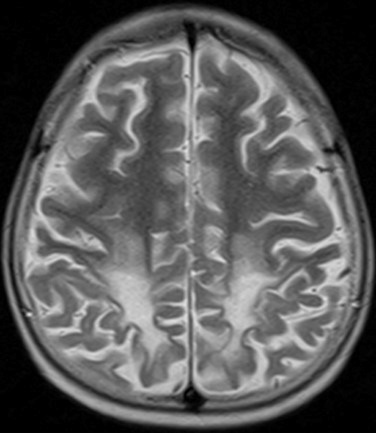

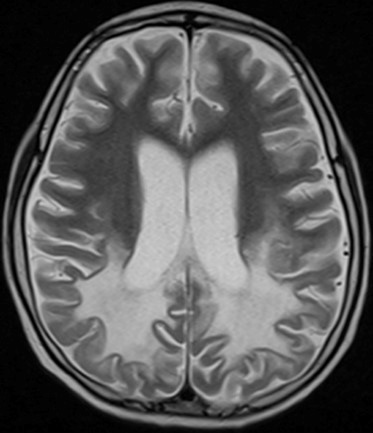

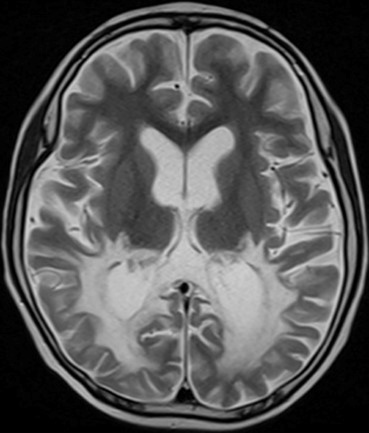


**A B C**


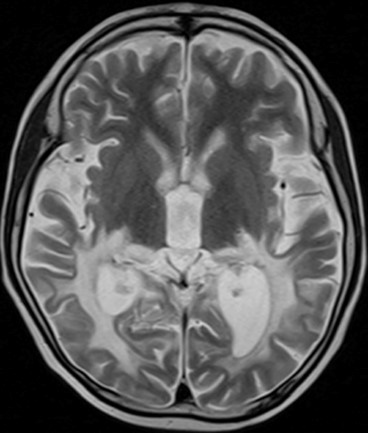

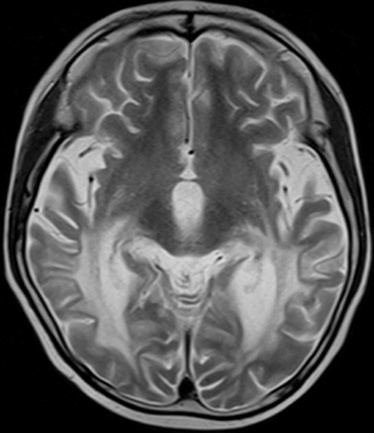

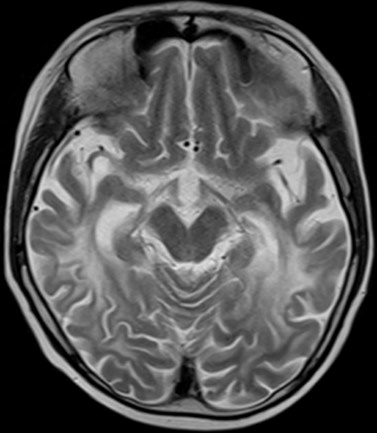


**D E F**


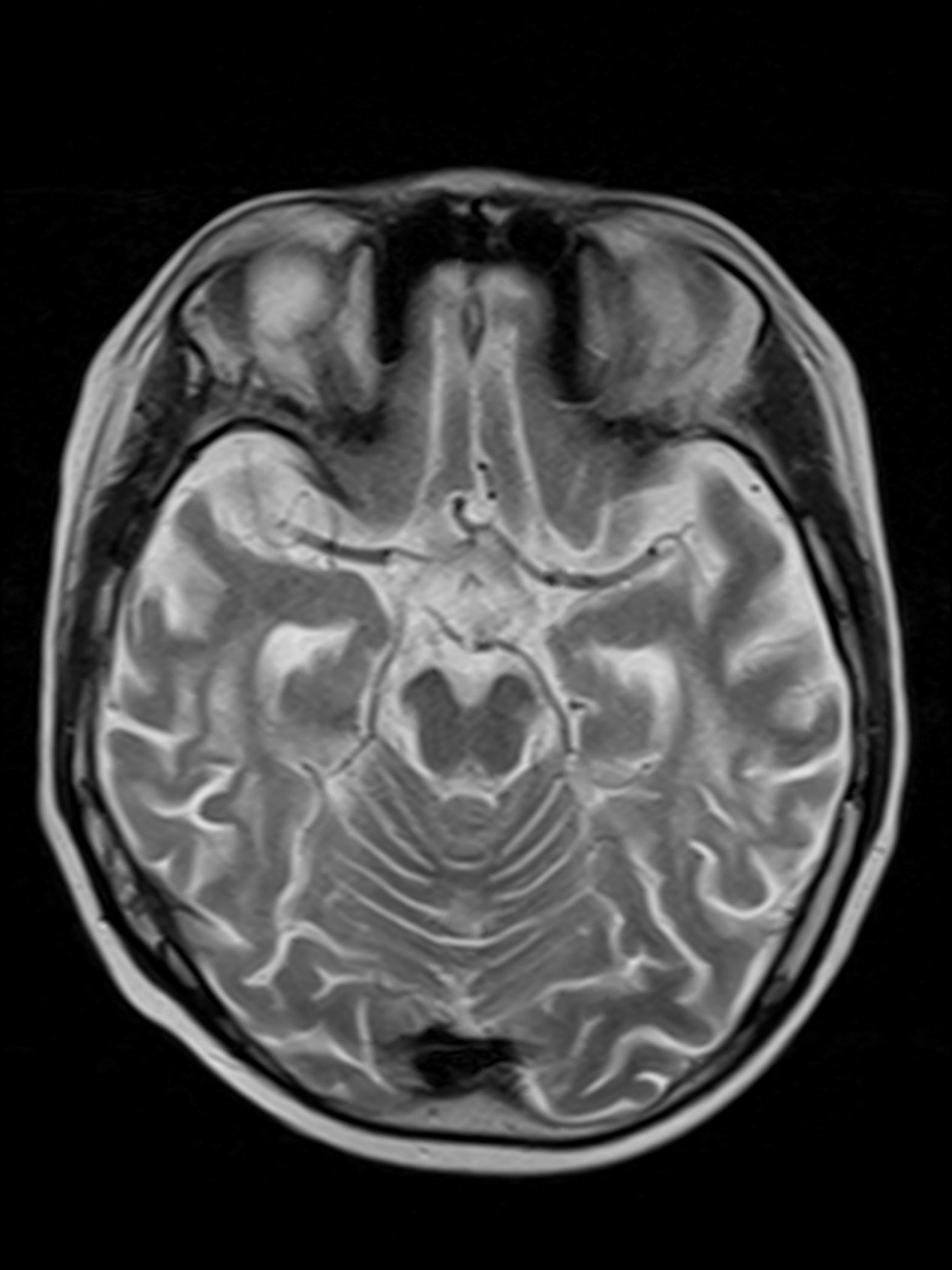

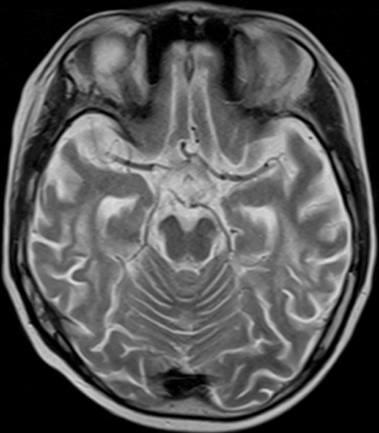

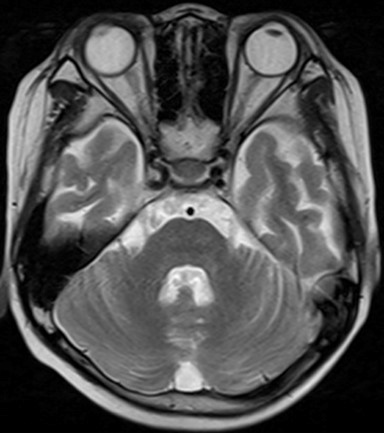


**G H**


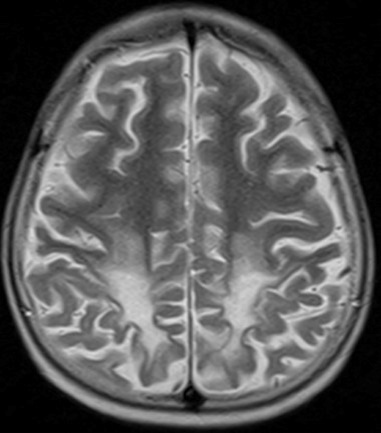

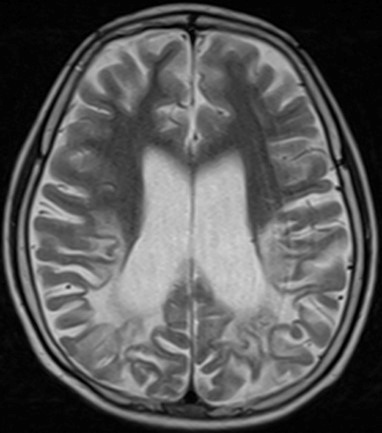

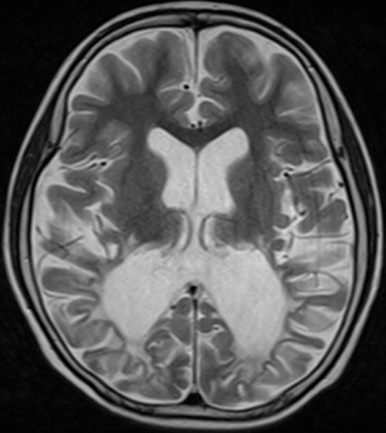


**I J K**


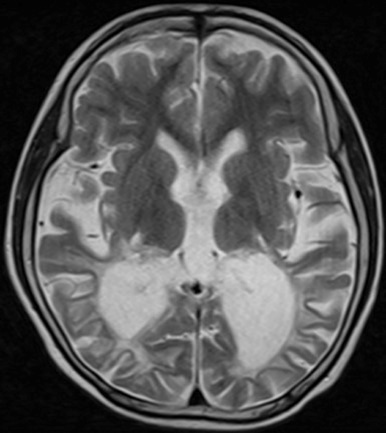

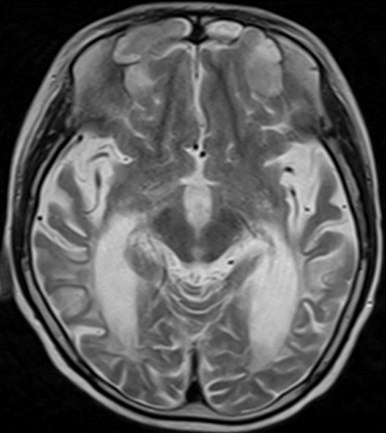

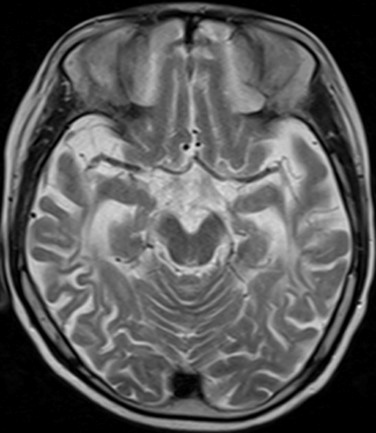


**L M N**


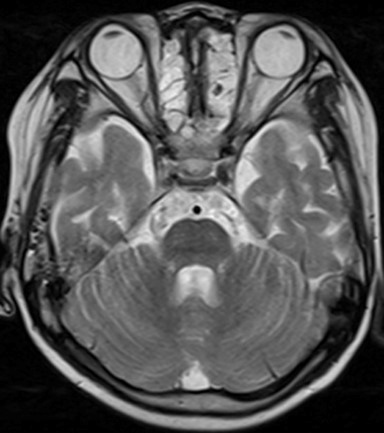


**O**

**Supplementary Figure 3.** MR images in a 9-year-old boy (P1) with adrenoleukodystrophy.

A-H. Pre-UCBT sequential axial T2 weighted images through brain from cephalad to caudal confirm involvement of the frontal, parietooccipital and anterior temporal white matter, corpus callosum, visual pathway, auditory pathway, internal capsule, basal ganglia, brain stem and cerebellum for a total MR severity score of 27.

I-O. Post-UCBT sequential axial T2 weighted images through brain from cephalad to caudal show the lesion areas are the same as pre-UCBT, but atrophy of the parietooccipital lobe and splenium of the corpus callosum is aggravated. The total MR severity score is 28.


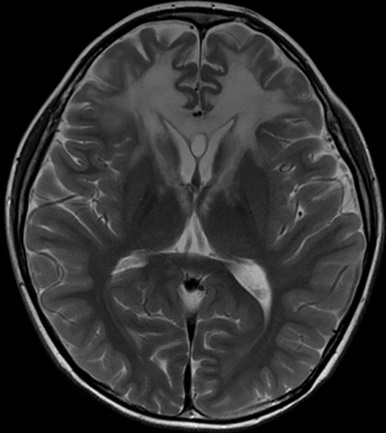

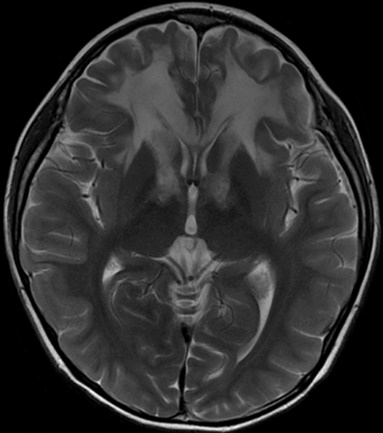

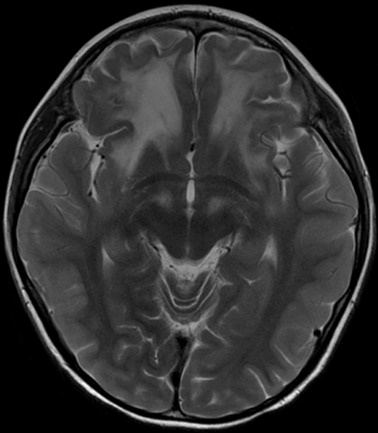


**A B C**


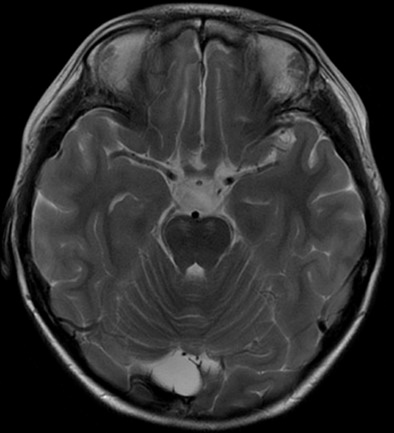


**D**

**Supplementary Figure 4.** MR images in a 7-year-old boy (P2) with adrenoleukodystrophy.

A-D. Sequential axial T2 weighted images through brain from cephalad to caudal confirm involvement of the frontal and anterior temporal white matter, corpus callosum, internal capsule, basal ganglia and brain stem for a total MR severity score of 11.


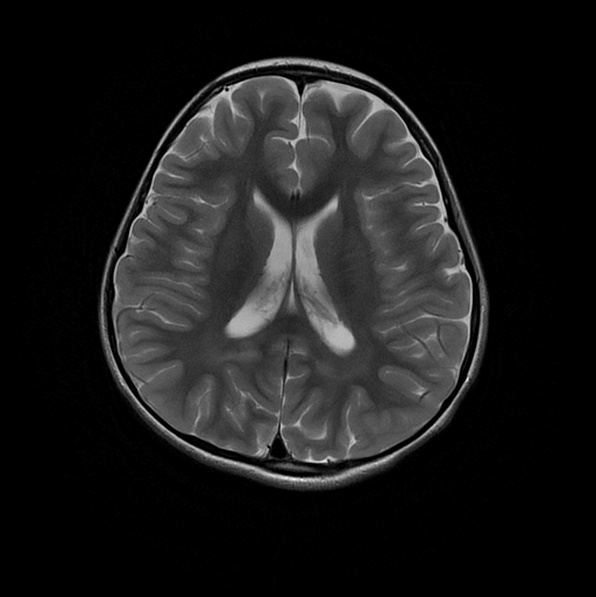

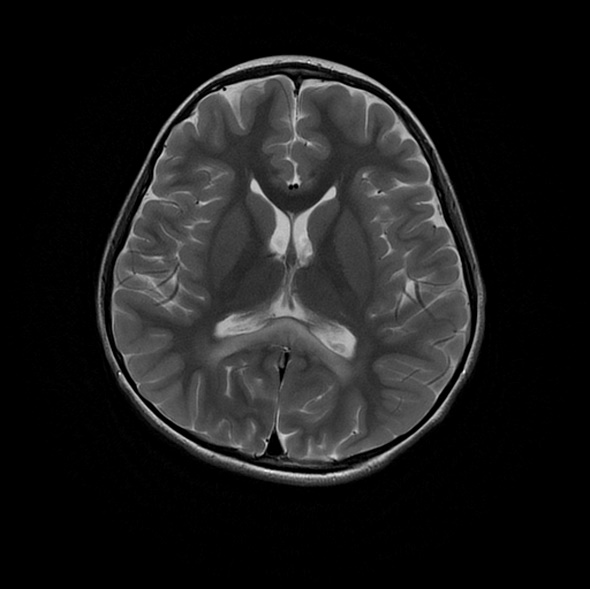

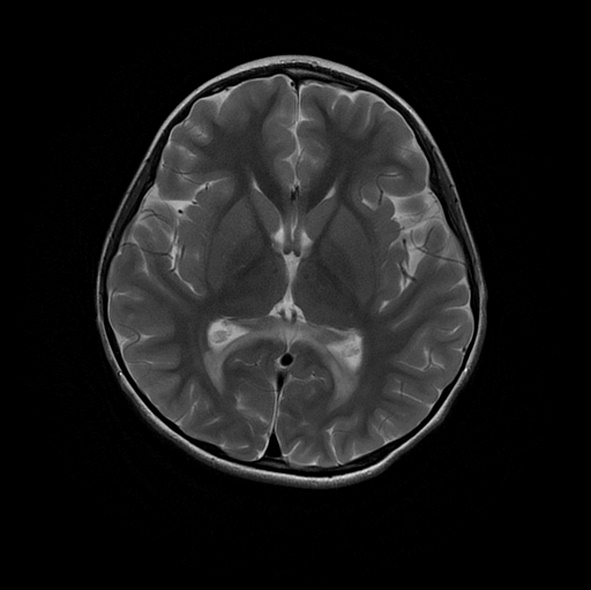


**A B C**


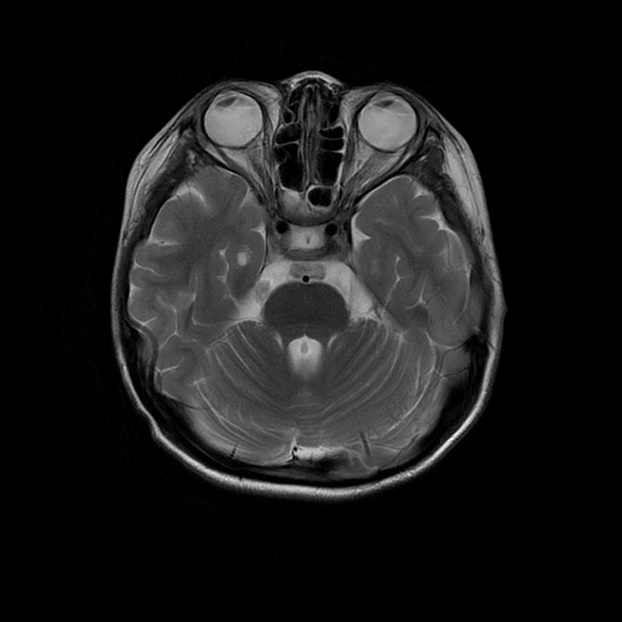

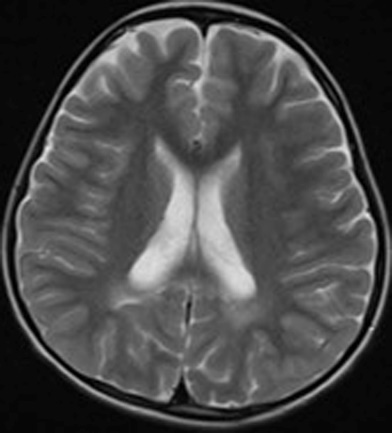

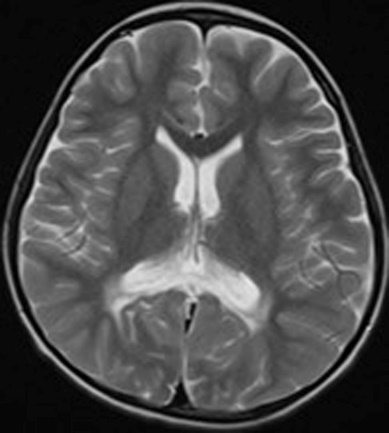


**D E F**


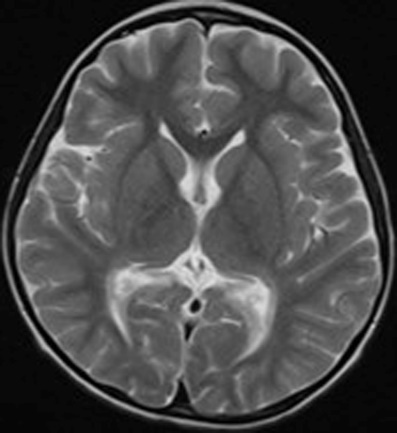

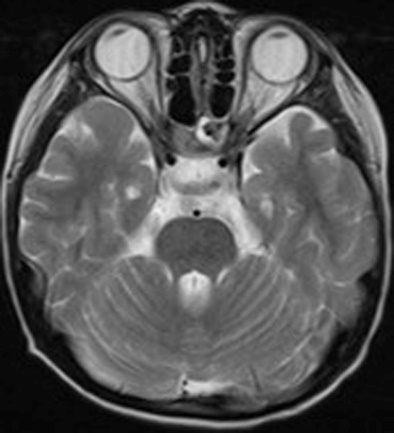


**G H**

**Supplementary Figure 5.** MR images in a 6-year-old boy (P3) with adrenoleukodystrophy.

A-D. Pre-UCBT sequential axial T2 weighted images through brain from cephalad to caudal confirm involvement of the parietooccipital white matter, splenium of the corpus callosum, and cerebellum for a total MR severity score of 3.

E-H. Post-UCBT sequential axial T2 weighted images through brain from cephalad to caudal reveal the lesion areas of the parietooccipital lobe are enlarged with local atrophy, the optical radiations are also involved. The total MR severity score is 7.


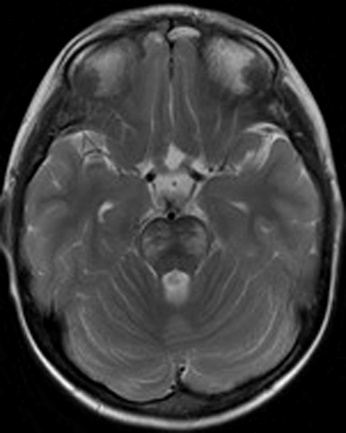

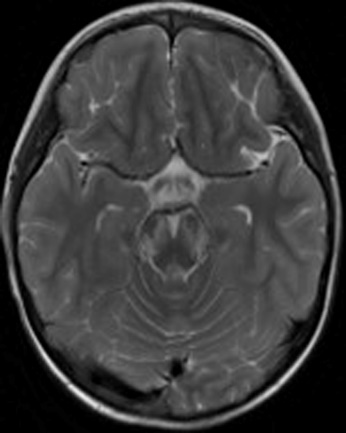

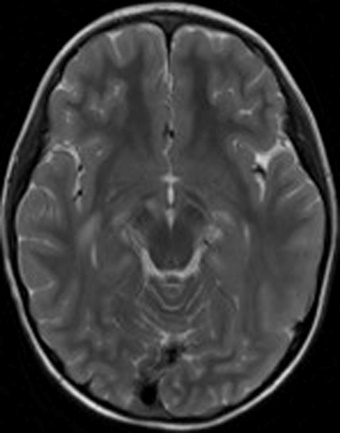


**A B C**


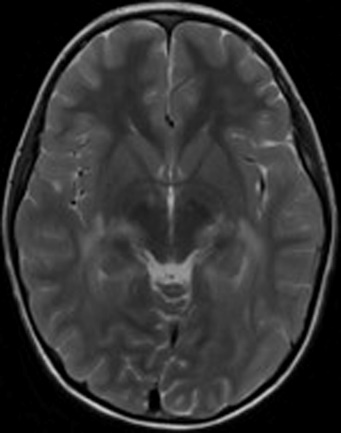

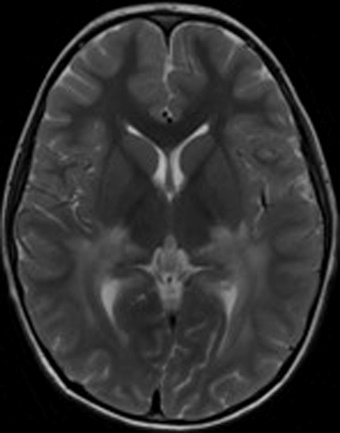

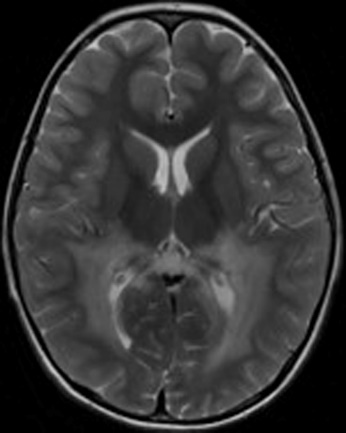


**D E F**


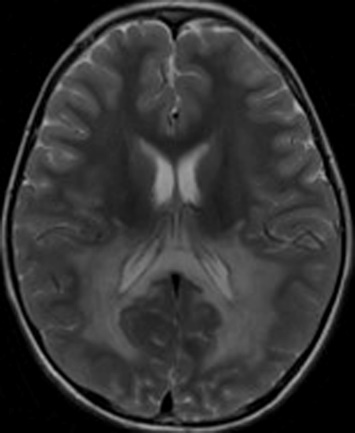

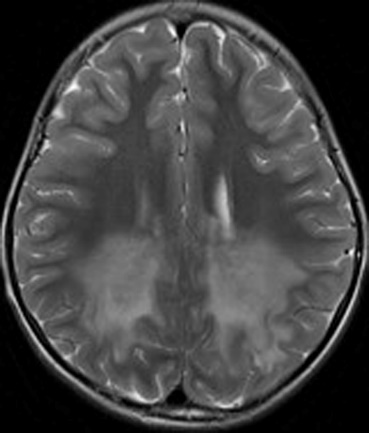


**G H**

**Supplementary Figure 6.** MR images in a 5-year-old boy (P4) with adrenoleukodystrophy.

A-H. Sequential axial T2 weighted images through brain from caudal to cephalad confirm involvement of the parietooccipital white matter, corpus callosum, visual pathway, auditory pathway, internal capsule, basal ganglia, brain stem and cerebellum for a total MR severity score of 15.5.


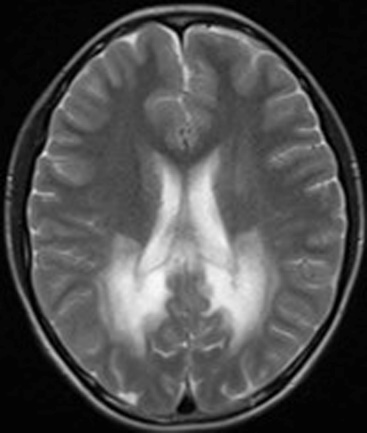

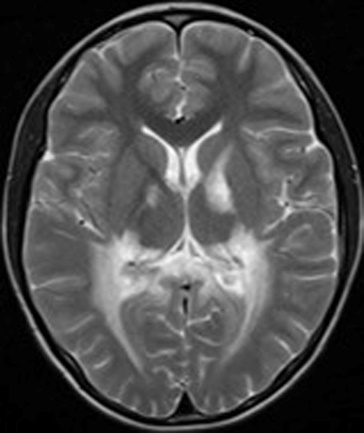

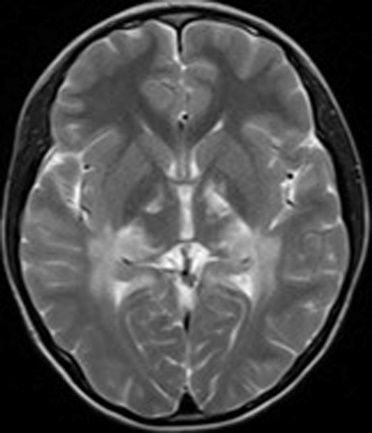


**A B C**


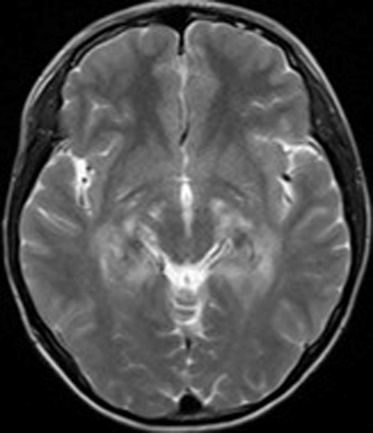

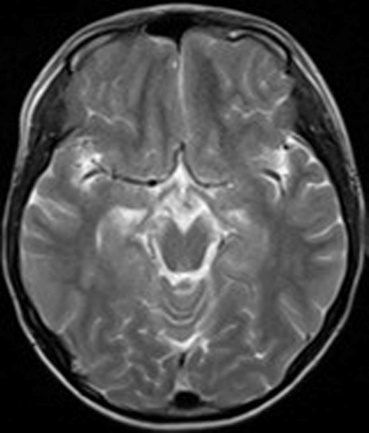

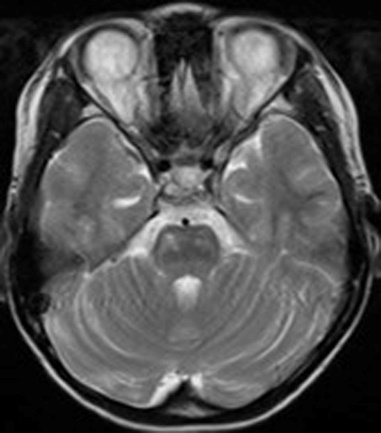


**D E F**


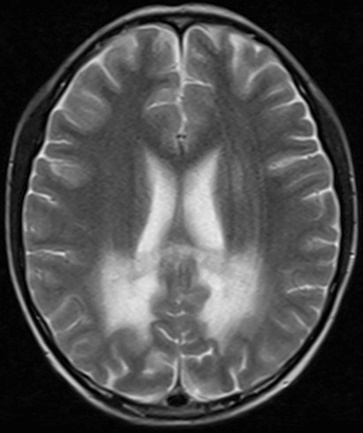

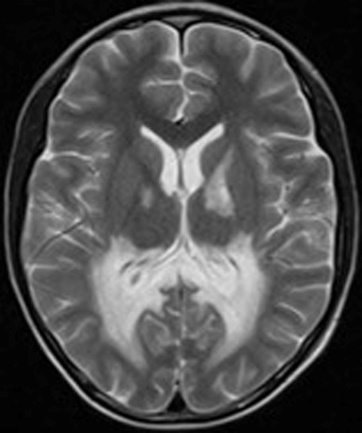

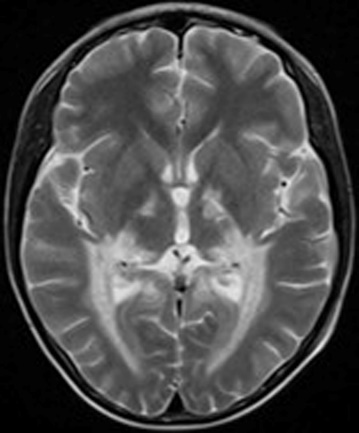


**G H I**


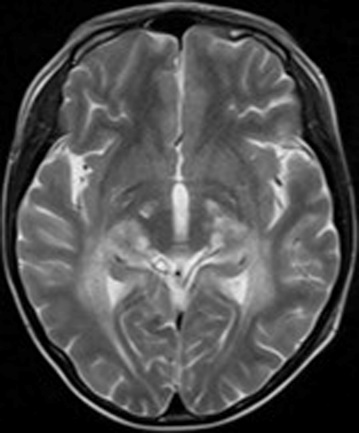

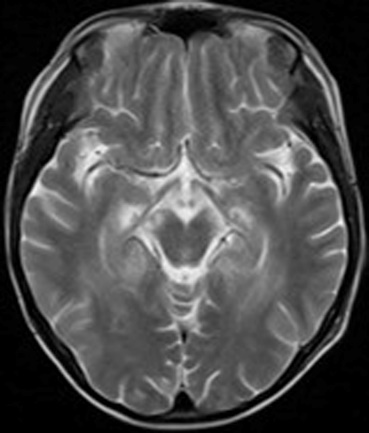

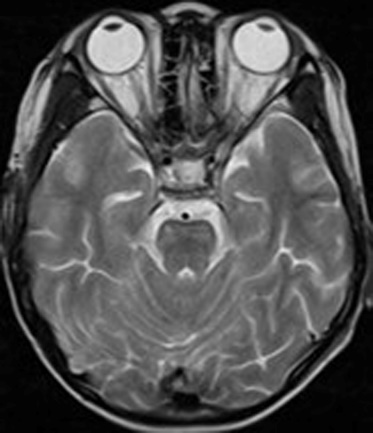


**J K L**

**Supplementary Figure 7.** MR images in a 12-year-old boy (P5) with adrenoleukodystrophy.

A-F. Pre-UCBT sequential axial T2 weighted images through brain from cephalad to caudal confirm involvement of the parietooccipital white matter, corpus callosum, visual pathway, auditory pathway, internal capsule, basal ganglia, brain stem and cerebellum for a total MR severity score of 18.

G-L. Post-UCBT sequential axial T2 weighted images through brain from cephalad to caudal show the atrophy of the frontal and temporal lobe in addition to the original lesions. The total MR severity score is 20.


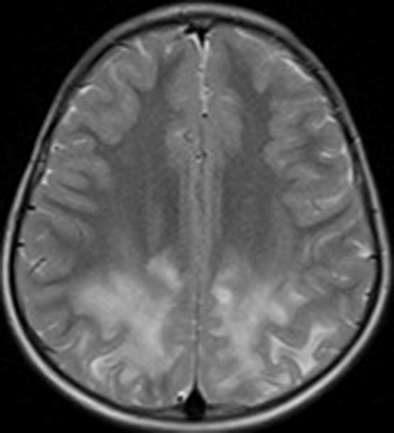

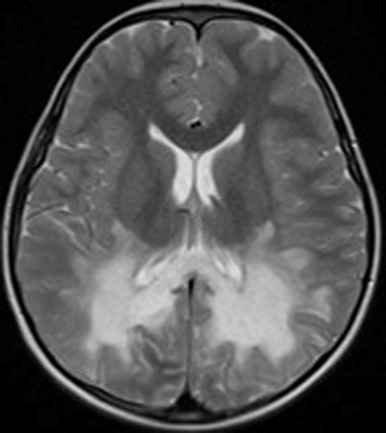

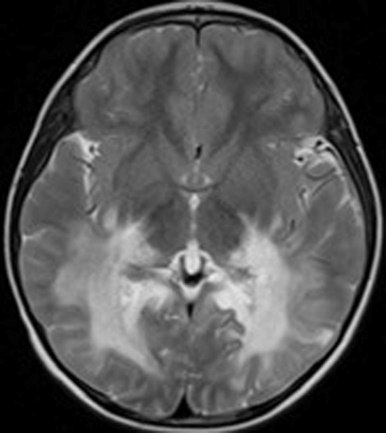


**A B C**


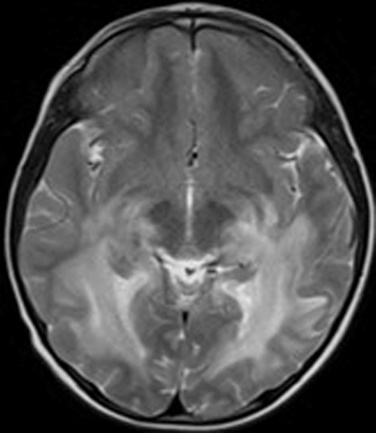

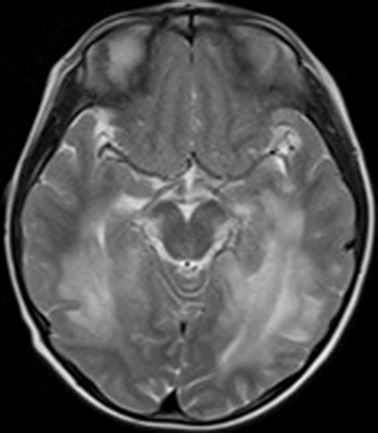

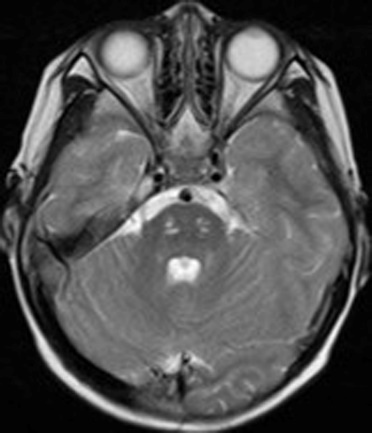


**D E F**


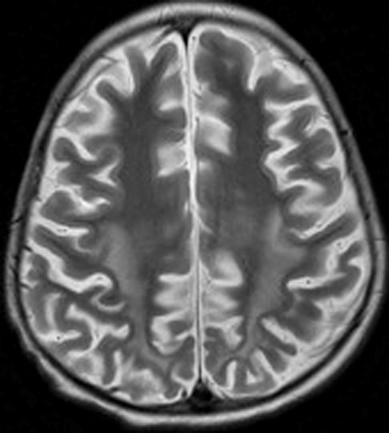

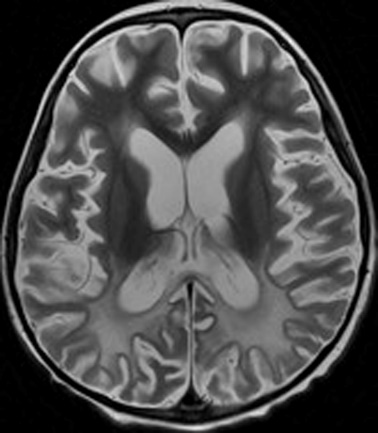

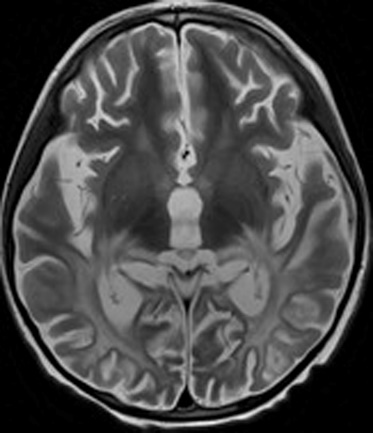


**G H I**


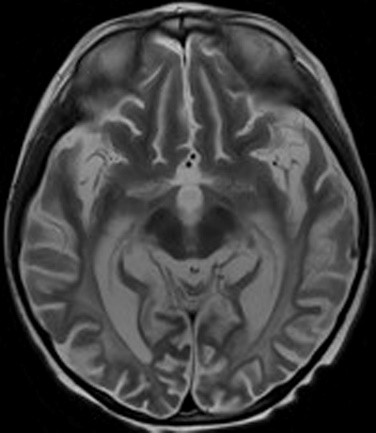

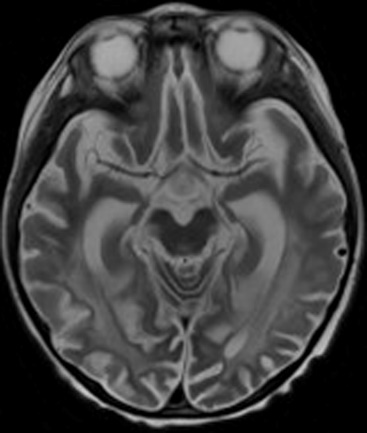

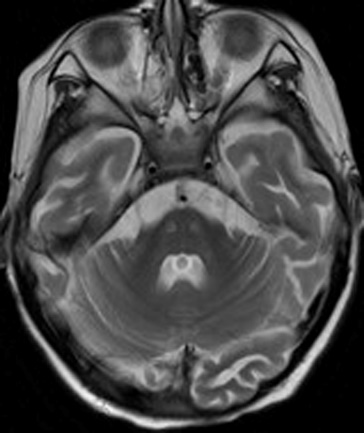


**J K L**

**Supplementary Figure 8.** MR images in a 4-year-old boy (P6) with adrenoleukodystrophy.

A-F. Pre-UCBT sequential axial T2 weighted images through brain from cephalad to caudal confirm involvement of the parietooccipital white matter, corpus callosum, visual pathway, auditory pathway, internal capsule, basal ganglia, and brain stem for a total MR severity score of 18.

G-L. Post-UCBT sequential axial T2 weighted images through brain from cephalad to caudal show significant enlargement of the lesion areas with brain atrophy. The total MR severity score is 30.


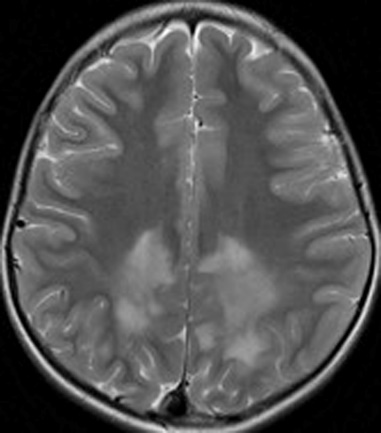

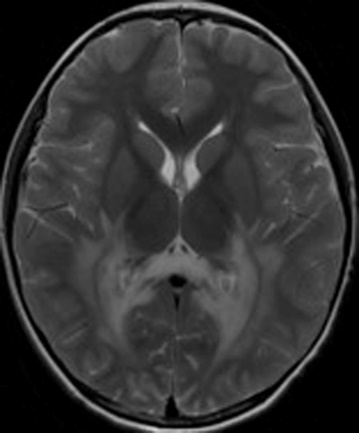

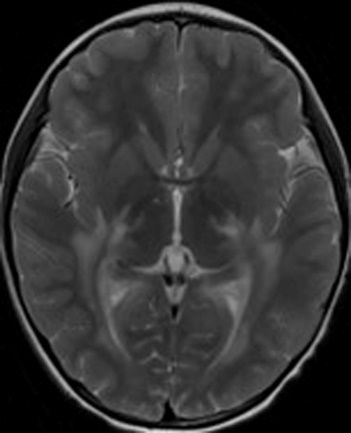


**A B C**


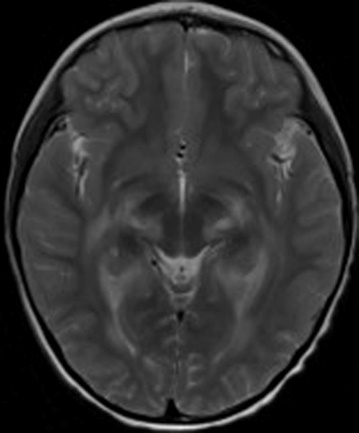

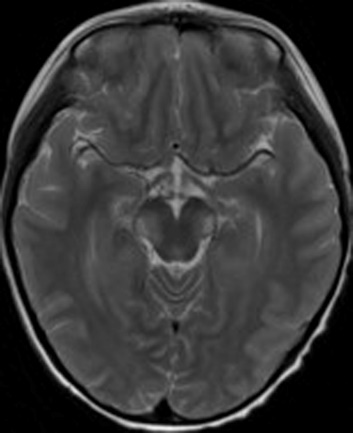

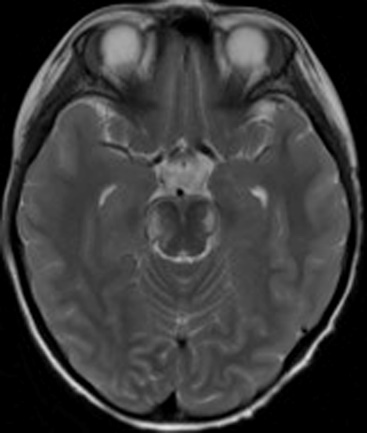


**D E F**


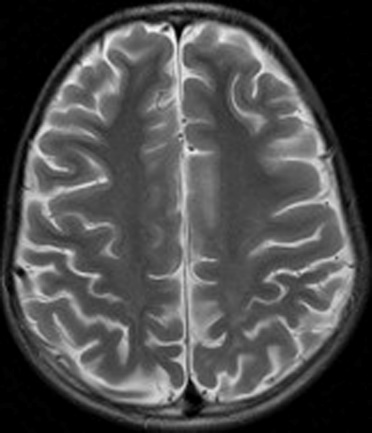

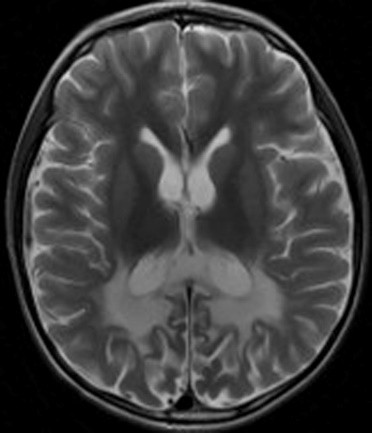

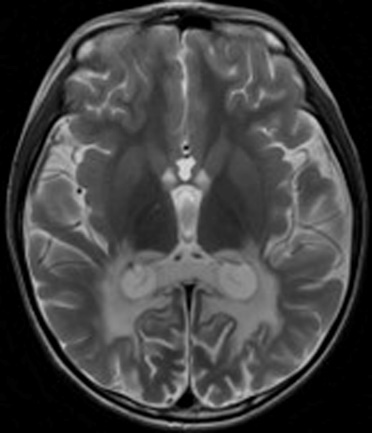


**G H I**


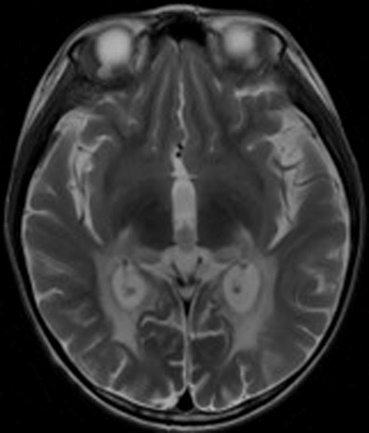

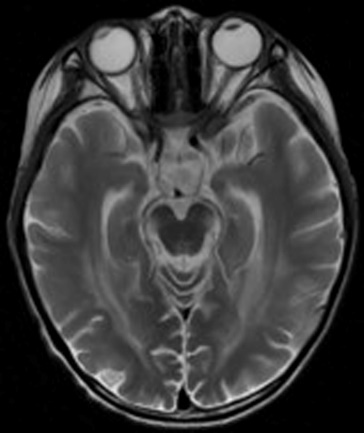

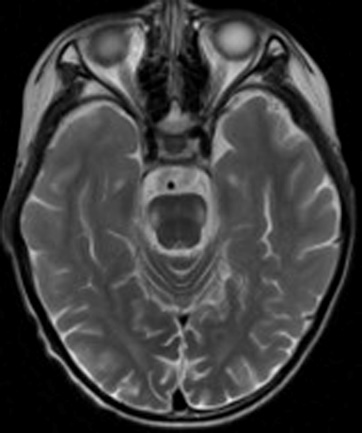


**J K L**

**Supplementary Figure 9.** MR images in a 6-year-old boy (P7) with adrenoleukodystrophy.

A-F. Pre-UCBT sequential axial T2 weighted images through brain from cephalad to caudal confirm involvement of the parietooccipital and frontal white matter, corpus callosum, visual pathway, auditory pathway, internal capsule, basal ganglia, and brain stem for a total MR severity score of 19.

G-L. Post-UCBT sequential axial T2 weighted images through brain from cephalad to caudal show involvement of the temporal lobe and cerebellum with brain atrophy in addition to the original lesions. The total MR severity score is 24.


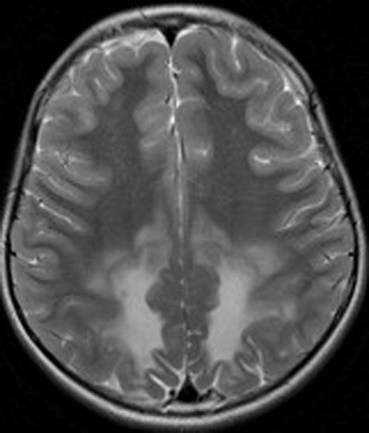

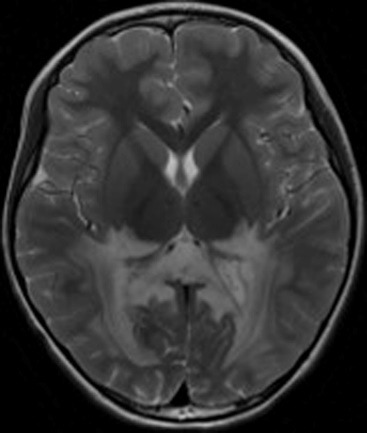

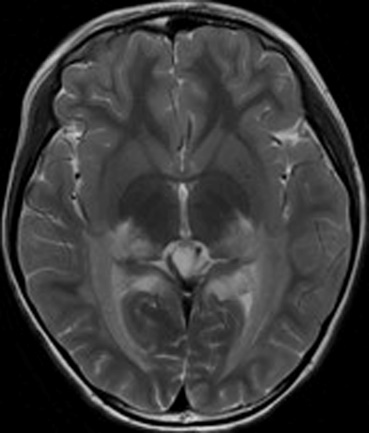


**A B C**


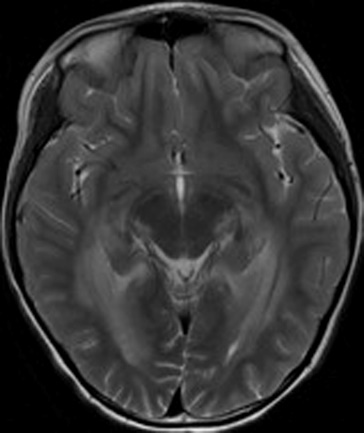

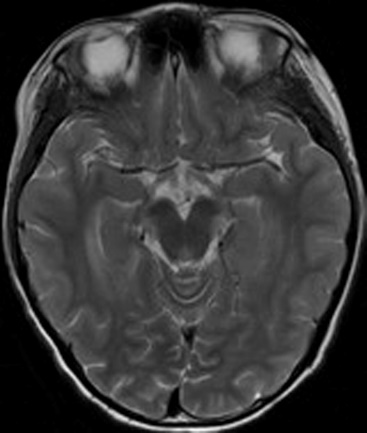

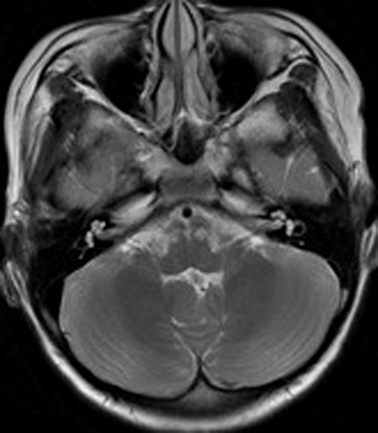


**D E F**


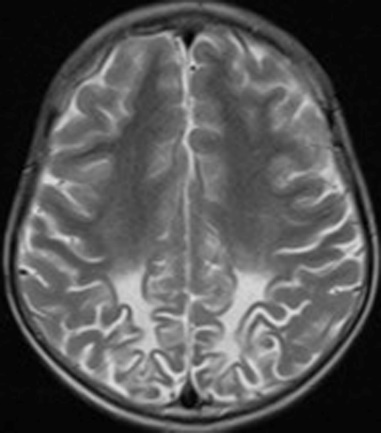

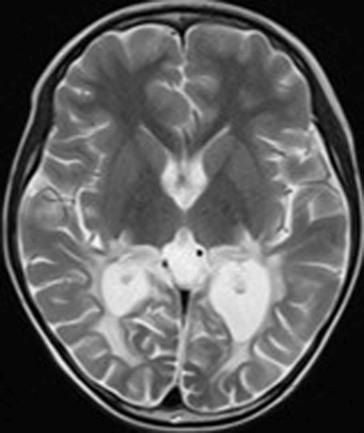

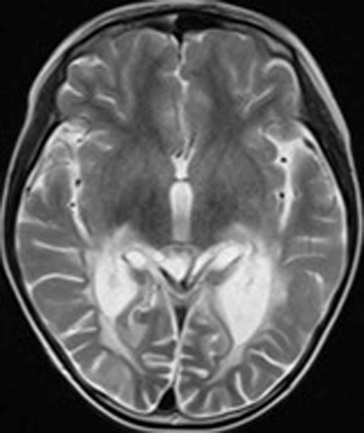


**G H I**


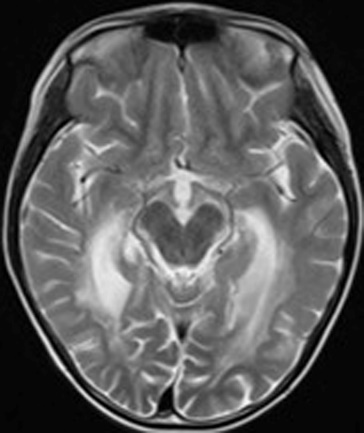

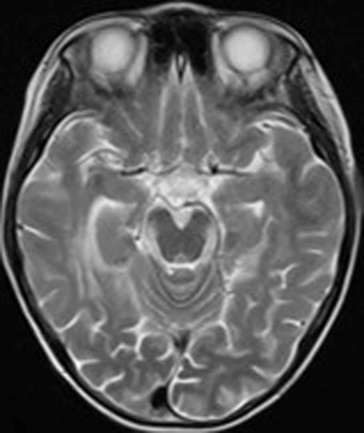

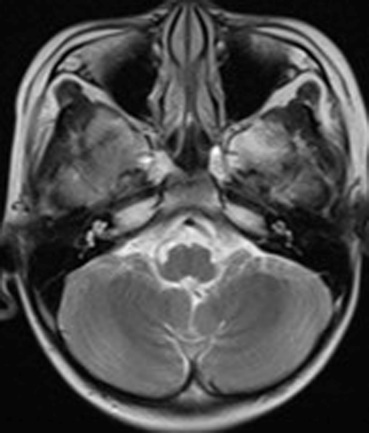


**J K L**

**Supplementary Figure 10.** MR images in a 8-year-old boy (P8) with adrenoleukodystrophy.

A-F. Pre-UCBT sequential axial T2 weighted images through brain from cephalad to caudal confirm involvement of the parietooccipital white matter, corpus callosum, visual pathway, auditory pathway, internal capsule, basal ganglia, and brain stem for a total MR severity score of 16.

G-L. Post-UCBT sequential axial T2 weighted images through brain from cephalad to caudal show improvement of the brain stem lesions but the brain is atrophy. The total MR severity score is 13.5.


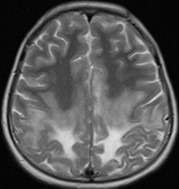

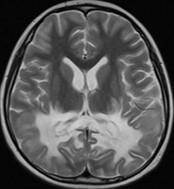

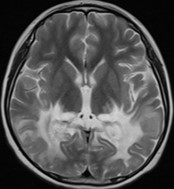


**A B C**


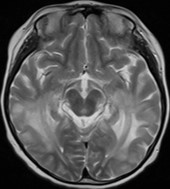

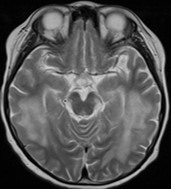


**D E**


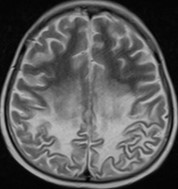

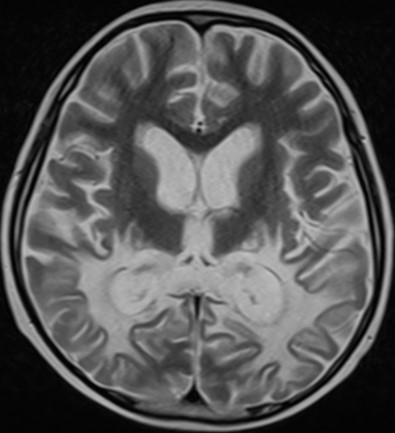

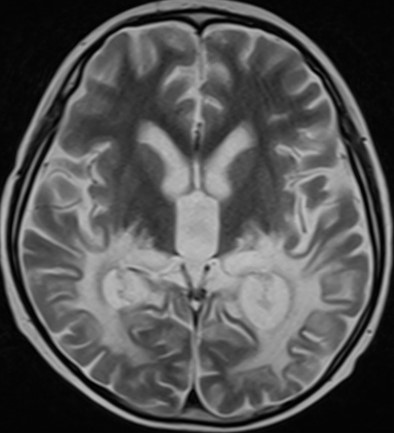


**F G H**


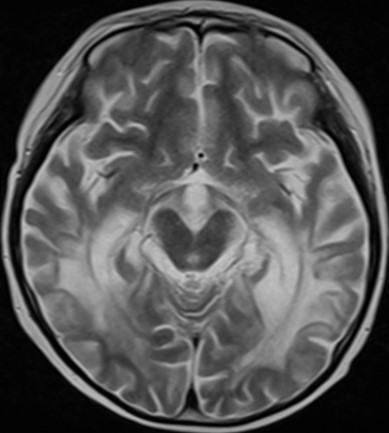

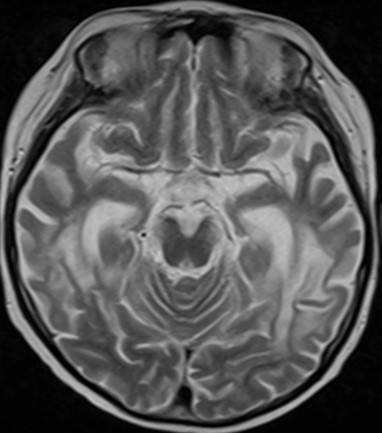


**I J**

**Supplementary Figure 11.** MR images in a 8-year-old boy (P9) with adrenoleukodystrophy.

A-E. Pre-UCBT sequential axial T2 weighted images through brain from cephalad to caudal confirm involvement of the frontal, parietooccipital and anterior temporal white matter, corpus callosum, visual pathway, auditory pathway, internal capsule, basal ganglia, brain stem with mild brain atrophy for a total MR severity score of 24.

F-J. Post-UCBT sequential axial T2 weighted images through brain from cephalad to caudal show moderate brain atrophy in addition to the original lesions. The total MR severity score is 27.


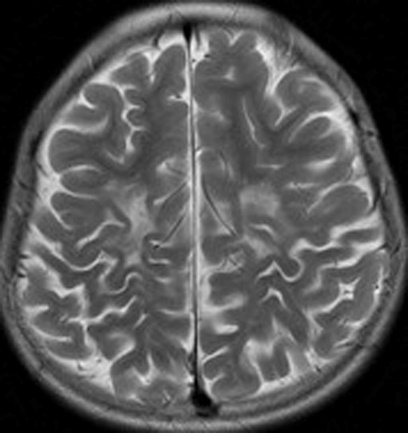

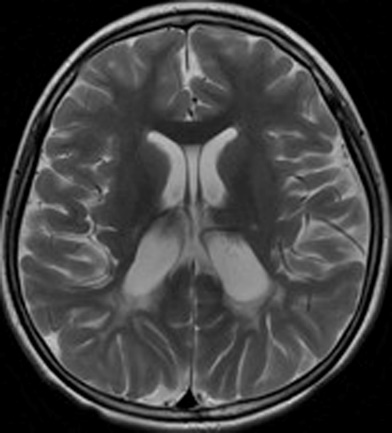

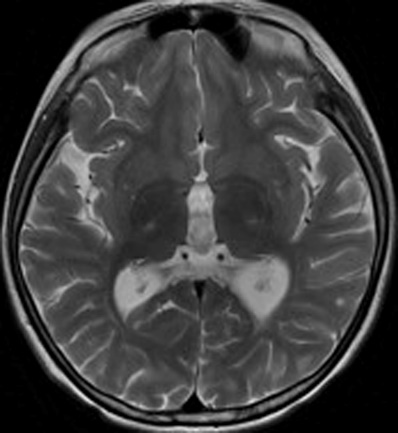


**A B C**


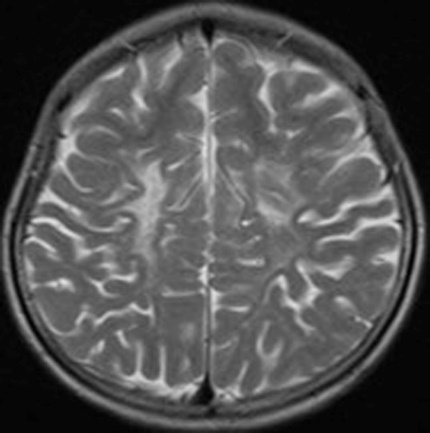


**D E F**

**Supplementary Figure 12.** MR images in a 7-year-old boy (P10) with globoid cell leukodystrophy.

A-C. Pre-UCBT sequential axial T2 weighted images through brain from cephalad to caudal confirm involvement of the frontal, parietooccipital and anterior temporal white matter, corpus callosum, pyramidal system with brain atrophy for a total MR severity score of 16.

D-F. Post-UCBT sequential axial T2 weighted images through brain from cephalad to caudal show the lesion areas and the MR severity score are the same as pre-UCBT.

**A B C**

**D E F**

**G H**

**Supplementary Figure 13.** MR images in a 3-month-girl (P11) with globoid cell leukodystrophy.

Pre-UCBT sequential axial T2 weighted images (A-D) and Post-UCBT sequential axial T2 weighted images (E-H) show the normal brain structures for a total MR severity score of 0.

**A B**

**C D**

**E F**

**G H**

**Supplementary Figure 14.** MR images in a 4-year-old boy (P12) with globoid cell leukodystrophy.

A-D. Pre-UCBT sequential axial T2 weighted images through brain from cephalad to caudal confirm involvement of the frontal and parietooccipital white matter, corpus callosum, pyramidal system, visual pathway and basal ganglia for a total MR severity score of 14.

E-H. Post-UCBT sequential axial T2 weighted images through brain from cephalad to caudal show enlargement of the corpus callosum lesion, improvement of the brain stem lesion and basal ganglia lesion. The brain is mild atrophy. The MR severity score is 16.
